# Trust-driven consensus reaching in human-AI hybrid large-scale group decision-making

**DOI:** 10.3389/frai.2026.1886098

**Published:** 2026-07-29

**Authors:** Xinyu Wang, Xuanhua Xu, Weiwei Zhang

**Affiliations:** 1 School of Advanced Interdisciplinary Studies, Hunan University of Technology and Business, Changsha, China; 2 School of Business, Central South University, Changsha, China

**Keywords:** decision consensus, human-AI collaboration, human-AI collective, human-AI trust, large language model

## Abstract

**Introduction:**

To address the issues of insufficient trust representation, lack of feedback in opinion conflicts, and low consensus convergence efficiency in human-AI hybrid group decision-making, this paper proposes a trust-driven consensus-reaching method for human-AI collaborative decision-making.

**Methods:**

Centered on trust modeling, the proposed method integrates human experts and large language models into a unified collaborative framework. By constructing dynamic trust relationships among multiple agents, it realizes the coupled evolution of trust mechanisms and opinion dynamics. Furthermore, a differentiated opinion updating mechanism is designed based on trust propagation, and combined with consensus measurement and feedback regulation to form an iterative process from initial opinions to a stable consensus solution.

**Results:**

Based on the Zhengzhou “7.20” rainstorm case, and in comparison with traditional weight adjustment methods and dynamic regulation methods based on deep reinforcement learning, the proposed method preliminarily validates its potential advantages in the above indicators.

**Discussion:**

Under the conditions of this case, the proposed method exhibits a trend of achieving higher consensus quality with lower intervention costs.

## 1 Introduction

In recent years, urban waterlogging has become an important type of disaster affecting urban public safety in China. According to statistics from the Ministry of Housing and Urban-Rural Development, more than 360 cities nationwide are facing waterlogging problems, with an average of over 1,200 waterlogging incidents occurring annually in the past decade, among which major waterlogging incidents causing casualties and economic losses exceed 40 per year on average. The frequency and complexity of natural disasters and other emergencies, such as extreme meteorological disasters, public health events, and environmental pollution accidents, have been increasingly posing significant impacts on social order and public safety. Against this background, emergency decision-making for urban waterlogging faces high uncertainty and strong time constraints. Traditional experience-based decision-making models are difficult to effectively support scientific judgment in complex situations (Xu et al., 2019, 2018). Since urban waterlogging involves multi-domain knowledge and multi-sectoral coordination, including meteorology and hydrology, municipal drainage, traffic management, and emergency rescue, the decision-making process has gradually evolved into a large-scale group decision-making (LSGDM) approach (Zeng et al., 2021; Xu, 2020; Xu et al., 2015). Although group decision-making can pool multi-source knowledge and improve decision comprehensiveness, the traditional model of independent expert analysis suffers from prominent problems such as difficulty in information integration and slow opinion convergence. Therefore, there is an urgent need to introduce intelligent auxiliary tools to enhance the efficiency and quality of group decision-making.

With the development of big data and artificial intelligence (AI) technologies, introducing algorithms and simulation methods into group decision-making processes to assist humans in forming collective intelligence has become an important research direction (Li, 2022; Ramón et al., 2023; Tang and Liao, 2021; Zuheros et al., 2023). In particular, large language models (LLMs), represented by ChatGPT and DeepSeek, trained on large-scale corpora, are capable of generating highly coherent and contextually relevant decision information, demonstrating significant advantages in complex semantic understanding and reasoning, and have shown application value in multiple domains (Wang and Zhang, 2024; Li et al., 2025; Zhuang et al., 2025). Relevant studies have shown that introducing LLMs into the decision-making process combines the analytical, logical, and inferential capabilities of AI with the experiential, intuitive, and systematic qualities of humans, greatly reducing decision uncertainty and risk (Huang et al., 2025; Shneiderman, 2020; Zhao et al., 2025).

However, despite the excellent performance of LLMs in certain domains, they still exhibit deficiencies in decision-making (Brady et al., 2025). Due to the need for enhanced explainability of AI decision-making, the issue of “hallucinations” inducing misinformation dissemination has emerged repeatedly (Kala et al., 2026; Wang et al., 2025). Algorithm-generated content has failed to directly meet decision-makers' demands for transparency and openness in a wide range of application scenarios (Papakyriakopoulos, 2021), leading to problems of algorithm bias and disuse (Bockstedt and Buckman, 2026; Zhao et al., 2024). Shrestha et al. (2019) analyzed the priority relationship between human decision-making and AI decision-making. Owing to the “black box” nature of algorithmic decision processes, managers are held accountable for the outcomes of algorithmic decisions; consequently, most corporate managers lack sufficient trust in AI decision-making and still prefer personal decision-making as their first choice. Existing studies have shown that the level of human-AI trust is a key factor influencing the effectiveness of collaborative decision-making and is directly related to the adoption and application of AI technologies (Wang et al., 2026; Everett et al., 2026). Although some scholars have explored methods for measuring human-AI trust in fields such as autonomous driving, shipping, and industrial manufacturing (Lv et al., 2021; Huang et al., 2020; Rahman and Wang, 2018), existing research is mostly based on static perspectives and fails to adequately characterize the dynamic evolution of trust during human-AI interaction. In fact, trust exhibits significant dynamism, constantly adjusting as tasks progress and information feedback is received (Hou et al., 2025). Therefore, there is an urgent need to construct a decision-making mechanism that can dynamically evaluate and regulate the human-AI trust relationship.

In summary, the pure rationality and high computational power of intelligent algorithms make AI a potential decision-maker superior to humans in many contexts, and LLMs are gradually transforming from tools into team members. However, large-scale human-AI collaboration decision-making mechanisms for major events still require systematic research, particularly in the area of dynamic trust-driven consensus reaching, where significant deficiencies remain. Therefore, against the backdrop of major event decision-making, this paper proposes a human-AI collaboration decision-making framework that integrates large language models and expert groups. By characterizing the human-AI trust relationship and guiding the opinion evolution process, the proposed framework aims to improve the efficiency of group consensus reaching and the quality of decision-making.

The main innovations of this paper are as follows: (1) Proposing a new human-AI collaboration paradigm that employs LLMs as collaborative decision-making subjects, and constructing a closed-loop decision-making framework that integrates LLM initial decision-making and bidirectional opinion calibration; (2) Developing a human-AI trust measurement model that integrates initial trust and dynamic trust, enabling dynamic characterization and regulation of the human-AI trust relationship, thereby overcoming the limitations of single static measurement and ensuring the reliability of human-AI trust; (3) Proposing a subgroup division method based on the dual thresholds of “trust degree and consensus degree,” and explicitly revealing the role of dynamic trust as the driving signal for differentiated regulation strategies. Unlike existing trust-based consensus methods where trust is merely used for weight assignment, and also unlike human-AI collaboration studies that focus solely on task allocation between a single human and a single AI, the dynamic trust in this paper is updated in real time in each iteration and directly determines the regulation strategies for four types of subgroups: high-trust subgroups adopt LLM guidance; low-trust subgroups suppress LLM influence; and trust-based subgroups trigger compromise adjustments through a direction deviation threshold, thereby enabling trust to actively regulate the consensus process. This closed-loop mechanism–encompassing trust perception, subgroup classification, differentiated regulation, and trust updating–makes trust no longer a background variable but a critical condition for consensus evolution.

## 2 Theoretical foundations

### 2.1 Human-AI collaborative system

**Figure 1:**
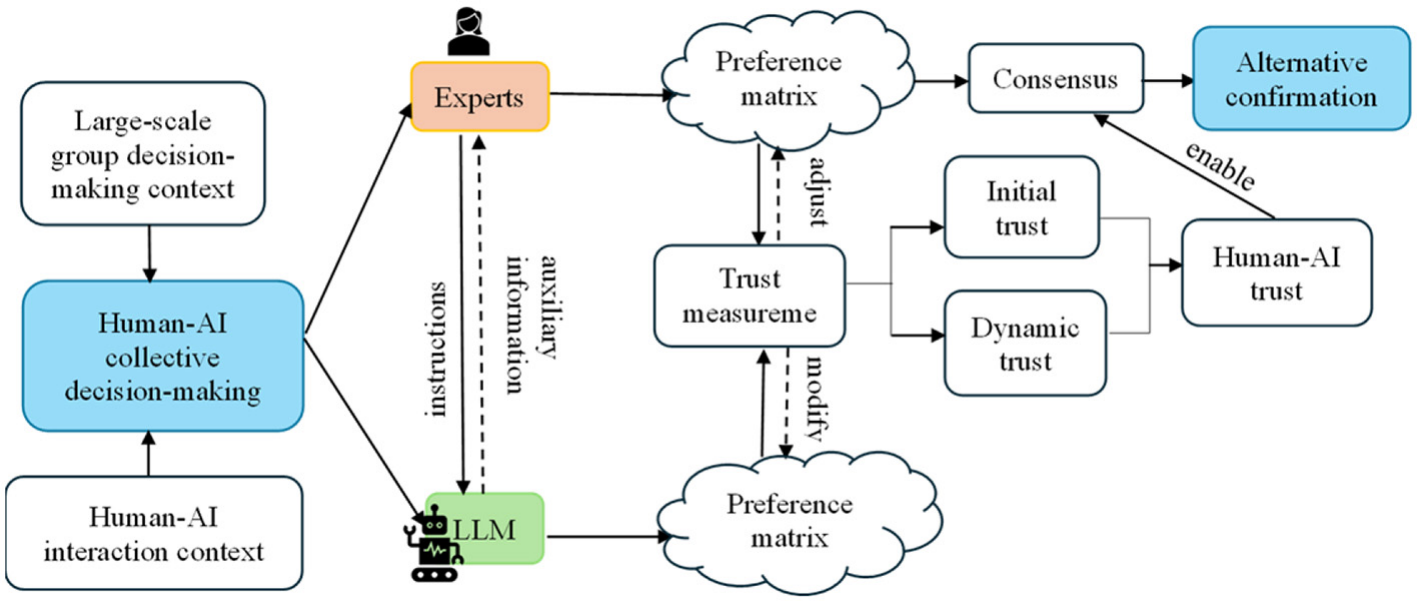
Human-AI group collaborative decision-making model.

Human-Machine Collaboration Theory is a theoretical framework aimed at exploring and optimizing how humans and intelligent machines, particularly artificial intelligence systems, can effectively combine and complement each other's strengths to accomplish complex tasks (Guo and Li, 2026). Its core idea is to transcend the traditional models of “humans using tools” or “machines assisting humans,” instead regarding humans and machines as a collaborative cognitive and decision-making system that emphasizes bidirectional interaction, dynamic adaptation, and intelligent integration (Jacucci et al., 2014). Humans excel at value judgment, contextual understanding, and creative trade-offs, while LLMs possess capabilities for massive data processing, multi-dimensional logical reasoning, and unbiased pattern recognition. Moreover, the cross-domain knowledge integration and high-dimensional reasoning processes of AI ultimately need to be transformed into decision outcomes through individual understanding, trust judgment, and adoption choices. Therefore, it is necessary to incorporate LLMs as subjects into the collaborative decision-making system to achieve a “1+1 > 2” effect. In the process of major event decision-making, first, experts confirm decision goals after deliberation; then, based on collaboration with LLMs, they obtain decision information; next, they measure human-AI trust by combining initial trust and dynamic trust, and adjust opinions according to human-AI trust. Finally, through consensus feedback and reaching processes, group opinions converge, and the final plan is determined. The human-AI collaboration decision-making framework is shown in Figure 1.

To standardize the research problem, the variables involved in the collaborative decision-making framework are described in advance. Consider a decision problem with *P* alternatives, denoted as *X* = {*x*_1_, *x*_2_, ..., *x*_*p*_}. These alternatives are evaluated against *N* attributes, denoted as *C* = {*c*_1_, *c*_2_, ..., *c*_*N*_}, the attribute weight vector is defined as *W* = {*w*_1_, *w*_2_, ..., *w*_*N*_}, satisfying $\overset{N}{\underset{j=1}{\sum }}w_{j}=1$. The decision-making bodies consist of human experts Ω = {*e*_1_, *e*_2_, ..., *e*_*M*_}(*M*≥20), along with a large language model L. The expert weight vector is defined as ω_*j*_ = {ω_1_, ω_2_, ..., ω_*M*_}, satisfying $\overset{M}{\underset{j=1}{\sum }}\omega_{j}=1$. Let $P\left(e_{i}\right)=\left(p_{lj}^{i}\right)$ represent the preference matrix of expert *e*_*i*_, where element ${\left(p_{lj}^{i}\right)}_{P\times N}$ denotes expert *e*_*i*_'s evaluation of attribute *c*_*j*_ in alternative set *x*_*l*_. The large language model's preference matrix is represented as a ${\left(p_{lj}^{i}\right)}_{P\times N}$ dimensional matrix.

### 2.2 Method for generating evaluation information of large language models

As a collaborative decision-making subject, the LLM does not rely solely on natural language prompts for open-ended generation. Instead, it performs targeted generation of solution evaluation information under structured task constraints. Based on a preset decision-making background, solution information, and attribute system, the LLM's semantic reasoning capabilities are transformed into a standardized evaluation matrix that can be used for collaborative decision-making computations through unified prompt templates, output boundary control, and consistency verification mechanisms.

Specifically, first, human experts input the background information of the research problem, descriptions of alternative solutions, and definitions of evaluation attributes into the LLM, guiding it to complete solution understanding and attribute analysis within a specific emergency decision-making context. As shown in Table 1, the LLM retrieves relevant knowledge fragments from its vast pre-trained corpus, associates related knowledge points through internal probabilistic modeling, and performs logical reasoning and comprehensive evaluation (Kumar, 2024). To reduce random fluctuations in output and enhance comparability among evaluation results of different solutions, fixed generation parameters are adopted for generating evaluation information, and the temperature parameter is kept consistent across evaluation rounds to avoid deviations in evaluation values caused by excessively high sampling randomness. At the same time, to mitigate the impact of randomness from a single generation on the results, multiple independent samplings can be performed for the same prompt, and the final evaluation result is determined by mean aggregation or majority agreement. When the deviation among multiple outputs exceeds a preset threshold, regeneration or manual review is triggered to improve evaluation stability.

**Table 1:** Prompt framework.

Contextual information	Alternative information	Attribute information
Please assume the role of an emergency management expert and understand the decision-making context, including service duration, deployment location, circumstances, problems to be addressed, and desired objectives.	There are four alternatives to be evaluated. Please comprehend their technical specifications, resource requirements, and expected outcomes one by one. Alternative 1: [Specific content]; Alternative 2: [Specific content]; Alternative 3: [Specific content]; Alternative 4: [Specific content].	Assuming you are an expert in the relevant field, please evaluate the four solutions and conduct a systematic comparative analysis based on the four key evaluation criteria.

In terms of output format, explicit structural constraints are pre-set on the LLM, requiring it to provide both numerical evaluations and brief explanations for each alternative solution under each evaluation attribute. The attribute evaluation value is limited to the interval [0,1], where 0 indicates the weakest performance of the solution on the corresponding attribute, and 1 indicates the optimal performance. If the model output falls outside this interval, it will be truncated according to predefined rules or the model will be required to regenerate the output.

In addition, to enhance the methodological rigor of the evaluation results, a consistency check mechanism is further established. On one hand, it examines the semantic consistency between the evaluation value and the explanatory information for the same solution, preventing contradictions between numerical judgments and textual justifications. On the other hand, it checks the magnitude of changes in the machine evaluation matrix across different rounds; if abnormal fluctuations occur without significant feedback input, the output is considered unstable and is corrected accordingly. Furthermore, it verifies whether the evaluations at the attribute level and the overall level maintain basic logical coherence, avoiding obvious mismatches between local attribute evaluations and overall judgments. After applying the above constraints, the machine evaluation matrix is finally integrated and used as the fundamental input for subsequent human-AI trust updates and group consensus reaching.

Although this paper controls the quality of LLM outputs through structured prompting, fixed generation parameters, and consistency checking mechanisms, LLMs may still produce hallucinations. To mitigate this risk, the following measures are taken in this paper: First, the prompt explicitly requires the LLM to “evaluate solely based on the provided background information and solution descriptions, without adding facts not supplied.” Second, for each attribute evaluation of each solution, the LLM is required to simultaneously provide explanatory text, facilitating experts in identifying obvious errors. Meanwhile, a group trust threshold is set; when the group trust level falls below this threshold, an LLM evaluation correction is forcibly triggered, which can to some extent filter out the influence of hallucinations.

## 3 Trust-driven group decision-making optimization model

### 3.1 Trust modeling

Human-AI trust is generally defined as the psychological tendency and behavioral choice of a human individual, under uncertain decision-making situations, to delegate partial decision-making authority or execution rights to an artificial intelligence system, based on cognitive judgments regarding the capabilities, behaviors, and predictability of outcomes of the intelligent system (Zhang et al., 2026). From the evolutionary perspective of the human-AI collaboration process, the trust structure can be further divided into two levels: initial trust and dynamic trust.

Among them, initial trust reflects the prior judgment formed by an individual before actual interaction with the system, primarily derived from factors such as their overall attitude toward the relevant technology, risk preference, and past experience. This trust largely determines whether human-AI collaboration can be triggered and accepted. When the level of initial trust is low, users may directly refuse to use the AI system, thereby blocking the initiation of the collaboration process.

However, even if individuals have similar levels of initial trust, their trust evolution paths may still diverge rapidly during actual interaction. Dynamic trust characterizes the process by which users continuously revise and update their trust levels based on system feedback performance, such as decision accuracy and stability, during sustained use. This process directly affects the stability and optimization potential of human-AI collaboration and is a key mechanism for achieving long-term effective cooperation.

Based on this, this paper uniformly models initial trust and dynamic trust, characterizing them respectively as the a priori basis for trust formation and the interactive update mechanism, thereby constructing a comprehensive trust evaluation framework that integrates both static cognitive preferences and dynamic feedback adjustments, so as to enhance the reliability and adaptability of human-AI collaboration decision-making.

#### 3.1.1 Human-AI initial trust

Initial trust refers to the prior trust level that experts hold toward large language models before the human-AI collaboration decision-making process begins. It is measured through historical trust and social network trust.

Historical trust reflects the impact of experts' past experience with AI tools on their current trust evaluation, and is measured using a scale for data collection and calculation. In the field of intelligent decision support system research, the human-AI trust scale has been widely validated and applied (Jian et al., 2000). In this study, before the decision-making process begins, a seven-point Likert scale (ranging from “strongly disagree” to “strongly agree”) is used to collect experts' historical trust evaluations of the AI system, as shown in Table 2.

**Table 2:** Human-AI trust scale items.

Measurement item	Survey item
Perceived reliability	I frequently use large language models in my past experiences.
	I perceive the responses from large language models as reliable.
	Large language models consistently provide the suggestions I require.
Perceived technical competence	The decision outputs of large language models outperform my own decisions.
	Large language models appropriately utilize the information I provide.
	The decisions generated by large language models are comparable to those made by highly competent individuals.
Perceived understandability	I know how to formulate queries for large language models to obtain needed responses.
	I can comprehend the decisions made by large language models.
Trust	When uncertain, I tend to trust large language models over my own judgment.
	I often consider using large language models when making decisions.

Social network trust characterizes the extent to which experts form or adjust their trust in the AI system by being influenced by the opinions of other trusted experts within their social network, using social network analysis (SNA). SNA is commonly used to study trust relationships among members in a social network structure and the mechanisms by which conflict behaviors are influenced by the network structure (Liang et al., 2022). Modeling trust and influence relationships among decision experts from different domains and incorporating the impact of social networks in consensus repair enables more accurate identification of key opinion leaders and isolated nodes. Based on this, Definition 1 (Li et al., 2022) and Definition 2 (Zhang et al., 2018) are provided.

Definition 1 A social network structure can be denoted as *G*(Ω, *V*), where Ω = *e*_1_, *e*_2_, ..., *e*_*M*_ represents the set of nodes, and *V* = (*e*_*l*_, *e*_*k*_)|*e*_*l*_, *e*_*k*_ ∈ Ω, *l* ≤ *k* denotes the set of edges connecting these nodes. Within a decision-making context, nodes correspond to experts, and edges indicate trust relationships among them.

Definition 2 In the social network *G*(Ω, *V*), the weight of the edge between nodes *e*_*l*_ and *e*_*k*_ is expressed as μ ∈ [0, 1], which signifies the degree of trust between experts *e*_*l*_ and *e*_*k*_.

Integrating social network analysis to assess experts' trust in large language models and reaching consensus based on this can reduce the uncertainty of large language models, thereby making their decision results possess a certain degree of explainability.

The calculation formula for experts' historical trust *HT*_*i*_ is presented in Equation 1, where *in*_*i*_ represents the score of the *i*-th item in the scale, and *n* denotes the total number of items.


$$\begin{array}{l} HT_{i}=\frac{\sum_{i=1}^{10}in_{i}}{7n} \end{array}$$ (1)

Treating the weight of the edge between nodes as the trust relationship between them, an expert's trust in the machine propagates through these inter-node trust connections. The formula for calculating the propagated trust *ST*_*i*_ is given in Equation 2.


$$\begin{array}{l} ST_{i}=\frac{\sum_{j=1}^{n}\mu_{ij}\cdot {\left(-1\right)}^{k}\cdot HT_{j}}{n}\cdot \beta \end{array}$$ (2)

Where, when *HT*_*j*_≥0.5, let *k* = 0, when *HT*_*j*_ < 0.5, let *k* = 1. Here, μ_*ij*_ denotes the weight of the edge between nodes *i* and *j* in the social network, and β represents the trust propagation factor, which characterizes the effect of trust transmission within the social network, with β ∈ [0, 1]. The trust threshold is set to 0.5. When the human-AI trust level < 0.5, it indicates low expert trust in the machine, and the node propagates negative trust throughout the network. When the human-machine trust level ≥0.5, it signifies high expert trust in the AI, and the node disseminates positive trust across the network.

Finally, to ensure that the initial trust and the subsequent dynamic trust maintain a unified dimension, and that the human-AI trust degree always falls within the interval, a convex combination is adopted to fuse historical trust and social network trust, obtaining the expert's initial trust in the AI system, as shown in Equation 3.


$$\begin{array}{l} INT_{i}=\eta \cdot HT_{i}+\left(1-\eta \right)\cdot ST_{i} \end{array}$$ (3)

Where η is the weight of historical trust, and (1−η) is the weight of social network trust.

#### 3.1.2 Human-AI dynamic trust

Dynamic trust is updated based on preference differences. In each round of consensus iteration, the human-AI dynamic trust *DT*_*i*_ of expert *e*_*i*_ is reflected through the real-time changes in the output matrices of both parties and the dynamic update of the normalized mean absolute deviation, as calculated in Equation 4, where $v_{j}^{i}$ is the preference vector in the expert's preference matrix, and *m*_*j*_ is the evaluation vector in the machine evaluation matrix.

Specifically, each iteration begins with the LLM adjusting its output based on expert feedback, such as optimization suggestions or revised solutions, while experts adjust their own preferences based on the LLM's performance, resulting in quantifiable matrices from both sides. The changes in these matrices essentially reflect the re-evaluation of each other's capabilities and reliability by humans and the LLM. After treating the matrices as vectors in a high-dimensional space, the normalized mean absolute deviation between the vectors becomes a quantitative indicator of trust level: the smaller the distance, the closer the outputs of both sides are to consensus, and the higher the trust level; conversely, a larger distance indicates lower trust. As the output matrices are continuously adjusted in each iteration, the normalized mean absolute deviation is updated in real time, directly mapping the dynamic evolution of trust. If the distance decreases, trust increases, promoting deeper collaboration. If the distance increases, a trust repair mechanism is triggered until a new consensus is reached, forming a closed-loop cycle of “interaction-adjustment-trust calibration.”


$$\begin{array}{l} DT_{i}=1-\frac{1}{P}\overset{P}{\underset{j=1}{\sum }}\sqrt{{\left(v_{j}^{i}-m_{j}\right)}^{2}} \end{array}$$ (4)

The paper provides a discussion on the boundary conditions of dynamic trust, It can be seen that $0\leq 1-\frac{1}{P}\overset{P}{\underset{j=1}{\sum }}\sqrt{{\left(v_{j}^{i}-m_{j}\right)}^{2}}\leq 1$, hence 0 ≤ *DT*_*i*_ ≤ 1. When the expert's preference is perfectly consistent with the LLM output, *DT*_*i*_ = 1; When they are completely opposite, the theoretical minimum value of *DT*_*i*_ is 0. In practice, *DT*_*i*_ is typically distributed in the range [0.5, 0.9]. The initial trust is obtained via a convex combination of historical trust (scale scores normalized to [0,1]) and social network trust (guaranteed to lie within [0,1] by the trust propagation formula), and thus also falls within [0,1]. The overall trust degree is *T*_*i*_ = α·*INT*_*i*_+(1−α)·*DT*_*i*_, where α ∈ [0, 1]. Therefore, the entire trust measurement system is self-consistent and bounded.

Finally, the initial trust and dynamic trust are combined to obtain the trust level of *T*_*i*_ expert *i* in the large language model, as calculated in Equation 5, where α is the trust weighting factor, and represents the proportion of initial trust and dynamic trust in the overall trust level, and α ∈ [0, 1].


$$\begin{array}{l} T_{i}=\alpha \cdot INT_{i}+\left(1-\alpha \right)\cdot DT_{i} \end{array}$$ (5)

### 3.2 Consensus adjustment mechanism

In the process of large-scale group decision-making, when there are significant differences in evaluation results among experts or between humans and the AI system, it is necessary to revise the evaluation information through a consensus adjustment mechanism to gradually improve group consistency. In particular, when the evaluation results generated by a large language model are not accepted by the expert group, the evaluation information needs targeted optimization. Essentially, this process can be viewed as an iterative process of human-AI collaboration learning and feedback correction. To characterize the overall acceptance level of the expert group toward the LLM's evaluation results, the group trust level is defined as shown in Equation 6.


$$\begin{array}{l} GT=\frac{\sum_{i=1}^{M}T_{i}}{M} \end{array}$$ (6)

Suppose the group trust threshold is set as γ. When the group trust level falls below γ, it indicates that the experts have low trust in the current evaluation information generated by the LLM, and the evaluation revision mechanism needs to be triggered. To enhance the scientific rigor of the LLM evaluation revision mechanism, this paper provides theoretical support from two aspects: human feedback evaluation and feedback-driven optimization. Existing studies have pointed out that in complex specialized tasks, the output of large language models cannot fully rely on one-time generation results; human participatory evaluation can effectively identify model biases and improve result reliability (Tam et al., 2024). Therefore, when there is a deviation between the evaluation results generated by the LLM and expert judgments, revising the evaluation information at the attribute level or the overall level through expert feedback can gradually improve the consistency between the evaluation information and the group decision-making objectives, thereby enhancing the reliability and explainability of the model output. Furthermore, according to the sources and characteristics of evaluation deviations, the LLM evaluation revision is divided into the following two categories:

(1) Attribute-level evaluation revision. When experts can clearly identify a bias in the LLM's evaluation of a specific attribute of a particular solution, the local evaluation information needs to be adjusted. This type of revision emphasizes fine-grained correction at the single attribute dimension to improve the accuracy and interpretive consistency of the evaluation results.

(2) Overall evaluation revision. When the overall evaluation results generated by the LLM deviate significantly from the expert group's preference matrix, but the deviation cannot be easily attributed to specific attributes, a global adjustment of the evaluation results is required. This process aims to reduce the overall distance between the LLM's evaluation and the group consensus, while retaining the model's own reasonable judgment basis to avoid excessive homogenization.

To achieve the above two types of revision, this paper designs a unified prompt-guided framework, as shown in Table 3. By introducing expert feedback information and group preference constraints, the LLM is guided to perform structured optimization of the evaluation results while maintaining semantic consistency, thereby achieving efficient consensus convergence in human-AI collaboration.

**Table 3:** LLM evaluation and refinement prompt framework.

Modification type	Prompting
Attribute-specific evaluation refinement	Expert feedback integration request. Identified issue: the evaluation of Alternative 1 regarding Attribute 2 exhibits a deviation (underestimation/overestimation) from expert expectations. Requested action: recalibrate the evaluation metric for Alternative 1 specifically on Attribute 2; regenerate both the revised quantitative assessment and its corresponding explanatory rationale.
Holistic evaluation adjustment	Expert-informed model refinement request. Following the integration of expert feedback, the collective evaluation of the alternatives has been updated. Specifically regarding Alternative 1: Attribute 1: 0.7; Attribute 2: 0.8; … (additional attribute evaluations). Adjust evaluation outputs to demonstrate closer alignment with expert-provided values, maintain the original technical perspective where justified, and provide explicit rationale for specific adjustments and retained elements.

### 3.3 Subgroup collaboration mechanism based on “trust level-consensus level”

#### 3.3.1 Subgroup division model

In the process of large-scale group decision-making, participants generally have significant differences in knowledge structure, risk preference, and acceptance of AI. This heterogeneity often leads to opinion divergence and reduces the efficiency of consensus reaching. Traditional average adjustment or unified intervention strategies fail to fully reflect the behavioral characteristics of different members, which can easily impair consensus efficiency. Wheeler's (2008) group dynamics theory points out that subgroups naturally form within a group based on shared attitudes, goals, or identities. Effective group management should identify and leverage the dynamic characteristics of these subgroups rather than forcibly smoothing over differences. Meanwhile, social identity theory emphasizes that individuals gain a sense of identity and behavioral guidance by categorizing themselves into specific social groups, and this categorization profoundly affects their willingness to cooperate and behavioral patterns (Liu, 2024). Therefore, in the context of human-AI collaboration decision-making, it is necessary to identify and characterize the structural features within the group in order to implement differentiated consensus guidance strategies.

Based on the above ideas, this paper proposes a two-dimensional subgroup division method that integrates human-AI trust level and expert consensus level. This method simultaneously considers experts' trust in large language models and the proximity of expert opinions to the overall group preference, thereby more comprehensively characterizing experts' behavioral characteristics in collaborative decision-making. Among them, human-AI trust level reflects the degree of experts' recognition of the LLM's decision-making capability, while consensus level reflects the proximity of expert opinions to the overall group preference. The core idea is to use trust level and consensus level as key classification variables to more accurately identify the behavioral drivers and potential conflict points of different subgroups, thereby laying the foundation for implementing differentiated and theoretically sound intervention strategies.

Different from existing subgroup division methods that are based solely on consensus degree or static social network trust, the core of this paper lies in using the dynamically updated trust value from each iteration as the basis for division, enabling the subgroup types to evolve dynamically with the human-AI interaction process, thereby driving differentiated regulation strategies. Specifically, dynamic trust and consensus degree together constitute a two-dimensional feature vector, where dynamic trust is updated in each iteration by Equation 4 and reflects the real-time consistency between expert and LLM outputs. This design makes the regulation strategy no longer a fixed weight adjustment, but a trust-dependent, adaptive closed-loop control: the influence of the LLM is enhanced in high-trust subgroups, suppressed in low-trust subgroups, and the direction of opinion adjustment in trust-based subgroups is dynamically determined by the deviation threshold between dynamic trust and the group consensus direction (see Section 3.3.2).

**Figure 2:**
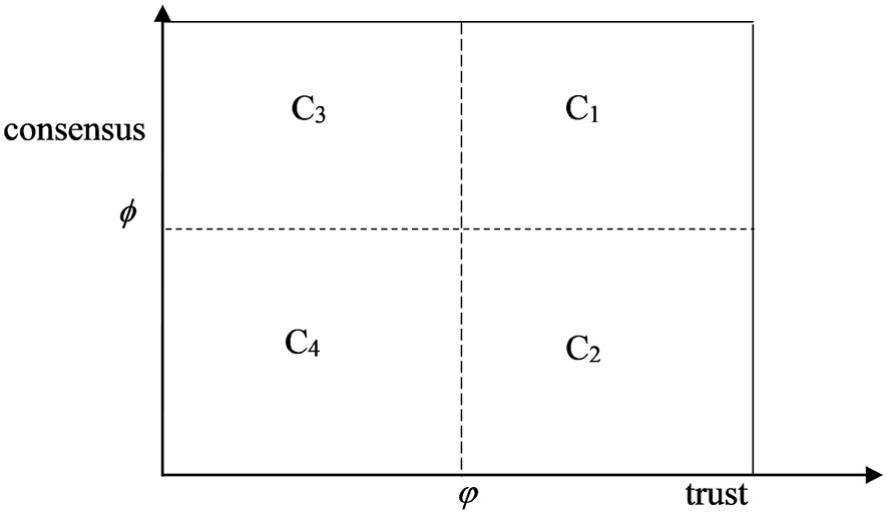
Human-AI group collaborative decision-making model.

Specifically, suppose the human-AI trust level of expert *e*_*i*_ is *T*_*i*_, and the consensus level between the expert's opinion and the group preference is *C*_*i*_. Let $v_{j}^{i}$ and $v_{j}^{E}$ be the preference vectors in the expert's preference matrix and the group preference matrix, respectively. Then the expert can be represented as a two-dimensional feature vector *S*_*i*_ = (*T*_*i*_, *C*_*i*_). Finally, according to Equations 7 and 8, and based on the trust-level threshold φ and the consensus-degree threshold ϕ, the large group is partitioned into four distinct categories (*C*_1_, *C*_2_, *C*_3_, *C*_4_), as illustrated in Figure 2.


$$\begin{array}{l} P{\left(p_{lj}^{E}\right)}_{P\times N}=\overset{M}{\underset{n=1}{\sum }}\omega_{i}\cdot {\left(p_{lj}^{i}\right)}_{P\times N} \end{array}$$ (7)

$$\begin{array}{l} Cl_{i}=\frac{1}{P}\overset{P}{\underset{j=1}{\sum }}dis\left(v_{j}^{i},v_{j}^{E}\right)=1-\frac{1}{P}\overset{P}{\underset{j=1}{\sum }}\sqrt{{\left(v_{j}^{i}-v_{j}^{E}\right)}^{2}} \end{array}$$ (8)

(1) Efficient subgroup

If *T*_*i*_ > φ and *Cl*_*i*_ > ϕ, then expert *e*_*i*_ belongs to the high-trust and high-consensus subgroup *C*_1_, referred to as the efficient subgroup. Experts in this subgroup both highly trust the large language model and maintain high consistency with the overall group opinion, making them a stable core group in collaborative decision-making. During the consensus evolution process, this subgroup is generally able to actively adopt reasonable suggestions and drive group opinions toward convergence.

(2) Trust subgroup

If *T*_*i*_ > φ and *Cl*_*i*_ < ϕ, then expert *e*_*i*_ belongs to the high-trust and low-consensus subgroup *C*_2_, referred to as the trust subgroup. Although, experts in this subgroup have high trust in the large language model, their personal preferences differ significantly from the group consensus. Such members tend to rely heavily on model suggestions for decision-making. Without reasonable guidance, their opinions may lead the group direction astray.

(3) Proximate subgroup

If *T*_*i*_ < φ and *Cl*_*i*_ > ϕ, then expert *e*_*i*_ belongs to the low-trust and high-consensus subgroup *C*_3_, referred to as the proximate subgroup. Although, such experts hold reservations about AI assistance, their opinions are relatively close to the overall group opinion. During the decision-making process, these members typically rely more on their own experience and have a lower degree of acceptance of model suggestions.

(4) Inefficient subgroup

If *T*_*i*_ < φ and *Cl*_*i*_ < ϕ, then expert *e*_*i*_ belongs to the low-trust and low-consensus subgroup *C*_4_, referred to as the inefficient subgroup. These experts neither trust the large language model nor align closely with the group opinion, making them a major factor affecting the efficiency of consensus reaching.

#### 3.3.2 Subgroup opinion management strategy

When the group consensus level ρ does not reach the group consensus threshold σ, it is necessary to adjust the opinions of experts within each subgroup. Based on Equation 9, the consensus level of subgroup *C*_*i*_ is calculated, where *n*_*i*_ is the number of members in subgroup *C*_*i*_, $v_{j}^{k_{1}}$ and $v_{j}^{k_{2}}$ represent the preference matrices of experts *e*_*k*_1__ and *e*_*k*_2__. The subgroup consensus levels are then aggregated to obtain the overall group consensus level, as shown in Equation 10. Based on this, different opinion management mechanisms are constructed.


$$\begin{array}{l} \rho_{i}^{C}=\frac{1}{C_{n_{i}}^{2}}\overset{n_{i}}{\underset{k_{1},k_{2}=1;k_{1}>k_{2}}{\sum }}\left(1-\frac{1}{P}\overset{P}{\underset{j=1}{\sum }}dis\left(v_{j}^{k_{1}}-v_{j}^{k_{2}}\right)\right) \end{array}$$ (9)

$$\begin{array}{l} \rho =\overset{4}{\underset{i=1}{\sum }}\frac{n_{i}}{M}\rho^{C_{i}} \end{array}$$ (10)

(1) Management of efficient subgroups and inefficient subgroups

Efficient subgroups are important driving forces for group consensus; therefore, intervention should be minimized during the consensus adjustment process, and a weight reward mechanism should be used to enhance their influence in group decision-making. In contrast, inefficient subgroups have a significant negative impact on consensus evolution, and a weight penalty mechanism is needed to reduce their interference in group decision-making.

The overall weight reward for efficient subgroups should be consistent with the overall weight penalty for inefficient subgroups. Suppose the weight reward coefficient is ζ^(*t*)^ and decreases as the number of rounds increases. The subgroup weights must satisfy the following conditions:


$$\begin{array}{l} \omega_{i}^{\left(t+1\right)}=\omega_{i}^{t}+\zeta^{t}\cdot \omega_{i}^{t},e_{i}\in C_{1} \end{array}$$ (11)

$$\begin{array}{l} \omega_{j}^{\left(t+1\right)}=\omega_{j}^{t}-\zeta^{t}\cdot \omega_{i}^{t}\cdot \frac{{n_{1}}^{t}}{{n_{4}}^{t}},e_{i}\in C_{1},e_{j}\in C_{4} \end{array}$$ (12)

Where $\omega_{i}^{\left(t\right)}$ and $\omega_{i}^{\left(t+1\right)}$ denote the weights of experts in rounds *t* and *t*+1 of the adjustment process, respectively; ${n_{1}}^{t}$ and ${n_{4}}^{t}$ represent the number of members in subgroups *C*_1_ and *C*_4_ during the *t*-th adjustment round, and ζ^(*t*)^ indicates the reward coefficient applied in round *t*.

(2) Trust subgroup management

**Figure 3:**
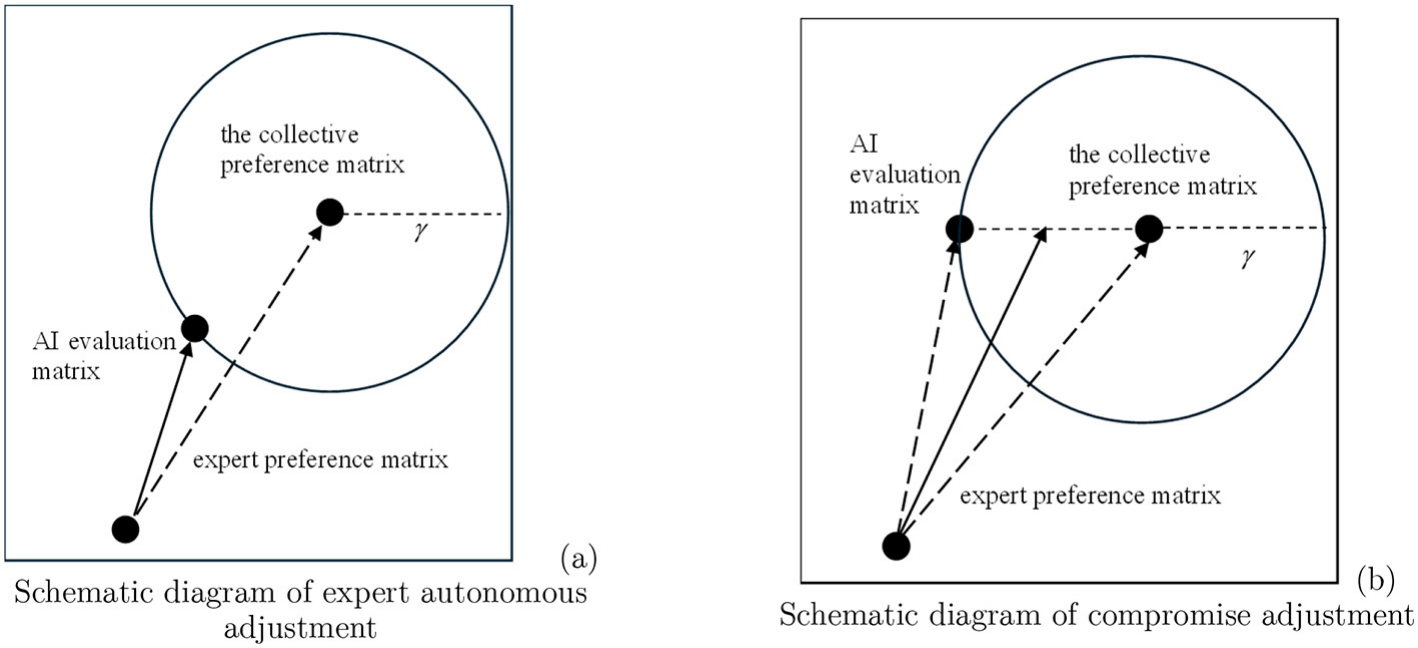
Trusted subgroup opinion steering diagram. Schematic diagram of **(a)** expert autonomous adjustment and **(b)** compromise adjustment.

Although trust subgroups trust the large language model, their opinions deviate from the group consensus and need to be guided to adjust toward the group consensus direction. First, determine whether the adjustment direction of the trust subgroup for each solution, based on the machine evaluation matrix, is close to the adjustment direction of the group preference matrix. When the expert's adjustment direction is consistent with the group consensus direction, autonomous adjustment may be permitted, as illustrated in Figure 3a.

If the adjustment direction for a particular solution deviates significantly, intervention in the opinion adjustment process is initiated. When the adjustment direction of the solution exceeds the directional deviation threshold θ, a compromise principle is adopted, allowing the expert to adjust along the median direction between their personal opinion and the group preference matrix. This preserves the expert's opinion to a certain extent while accelerating the consensus-reaching process, as illustrated in Figure 3b.

For alternative *j*, the angle θ_*ij*_ between expert *e*_*i*_'s opinion adjustment direction and the group's evaluation direction of the opinion is shown in Equation 13, where $v_{j}^{i}$,$v_{j}^{E}$,$v_{j}^{L}$ denote the evaluation vectors of expert *e*_*i*_, the expert group, and the large language model, respectively, concerning the *j*-th alternative.


$$\begin{array}{l} \theta_{ij}=cos^{-1}\left(\frac{\left|\left(v_{j}^{i}-v_{j}^{E}\right)\cdot \left(v_{j}^{i}-v_{j}^{L}\right)\right|}{\left|v_{j}^{i}-v_{j}^{E}|\cdot |v_{j}^{i}-v_{j}^{L}\right|}\right) \end{array}$$ (13)

(3) Proximate subgroup management

The proximate subgroup has low trust in the large language model but holds opinions similar to the group consensus. To achieve consensus quickly, excessive intervention is unnecessary; experts within this subgroup may modify their opinions autonomously. If an expert's preference revision goes against the direction of consensus reaching, the proposed revision is discarded, and the original preference matrix is retained.

## 4 Human-AI collective collaborative decision-making procedure

To facilitate the description of the human-AI collaboration consensus reaching process, this paper formalizes the method as a standardized decision-making procedure.

(1) Initial human-AI trust calculation

Before the decision-making process begins, historical trust data of experts toward the large language model are collected using a human-AI trust scale. At the same time, the transitive trust among experts is calculated based on the social network structure among them. The historical trust and social network trust are then integrated using Equations (1–3) to obtain the initial trust level of each expert in the large language model.

(2) Large language model generates initial evaluation information

According to the preset prompt template, the background information of the decision problem, the evaluation attributes, and the alternative solutions are input into the large language model. The large language model generates the evaluation results and their corresponding explanations for each solution based on the prompt information, and constructs the machine evaluation matrix.

(3) Experts generate initial preferences and compute dynamic trust

Based on the evaluation information and explanatory content generated by the large language model, experts assess each alternative solution to form an expert preference matrix. At the same time, Equation 8 is used to calculate the expert consensus level. Subsequently, based on the expert preference matrix and the machine evaluation matrix, the human-AI dynamic trust is calculated using Equation 4, and then combined with the initial trust to obtain the individual human-AI trust level of each expert. The group trust level of the expert group toward the large language model is then calculated by aggregating the individual trust levels.

(4) Human-AI trust level judgment and machine evaluation revision

A group trust threshold is set. When the group human-AI trust level falls below this threshold, it indicates that the expert group has insufficient trust in the evaluation information generated by the current large language model, and the LLM evaluation revision mechanism is triggered accordingly. The machine evaluation information is adjusted according to the prompt template, and the expert trust levels are recalculated until the group trust level meets the threshold requirement.

(5) Subgroup division and consensus level calculation

Once the group trust level meets the threshold requirement, the consensus reaching stage begins. Based on the expert's human-AI trust level and expert consensus level indicators, and using the trust threshold and consensus threshold, the expert group is divided into subgroups, forming efficient subgroups, trust subgroups, proximate subgroups, and inefficient subgroups. At the same time, the consensus level of each subgroup and the overall group consensus level are calculated.

(6) Subgroup opinion management and consensus adjustment

If the group consensus level does not reach the preset consensus threshold, differentiated opinion management strategies are adopted according to the behavioral characteristics of different subgroups. For efficient subgroups and inefficient subgroups, their influence in group decision-making is adjusted through weight reward and penalty mechanisms. For trust subgroups, an opinion adjustment direction guidance mechanism is used to facilitate their convergence toward the group consensus direction. For proximate subgroups, they are allowed to adjust their opinions autonomously within a certain range.

(7) Consensus convergence and alternative ranking

Through multiple rounds of opinion adjustment and weight updating, the group consensus level is gradually improved. When the group consensus level reaches the preset consensus threshold, the group consensus is considered achieved. At this point, the final group evaluation $g_{kj}^{t}$ of alternative *l* on attribute *j* after *t* iterations is obtained, as shown in Equation 14.


$$\begin{array}{l} g_{lj}^{t}=\overset{M}{\underset{i=1}{\sum }}\omega_{i}^{t}\cdot p_{lj}^{it} \end{array}$$ (14)

Where $\omega_{i}^{t}$ represents the expert weight after the *t*-th round of adjustment, and $p_{lj}^{it}$ represents the evaluation of expert *e*_*i*_ on alternative *l* under attribute *j* after *t* rounds of adjustment. Next, the expert preference matrices are aggregated. Since the elements of the preference matrices in this paper are all standardized cardinal evaluation values, and the attribute weights directly reflect the importance of each criterion. Therefore, the weighted average method is used to calculate the comprehensive score of each alternative, and the comprehensive score of the expert group for alternative *l* is shown in Equation 15. Combined with the attribute weights, the final ranking of alternatives is obtained, and the optimal decision alternative is determined.


$$\begin{array}{l} S_{l}=\overset{N}{\underset{j=1}{\sum }}w_{j}\cdot g_{lj}^{t} \end{array}$$ (15)

**Figure 4:**
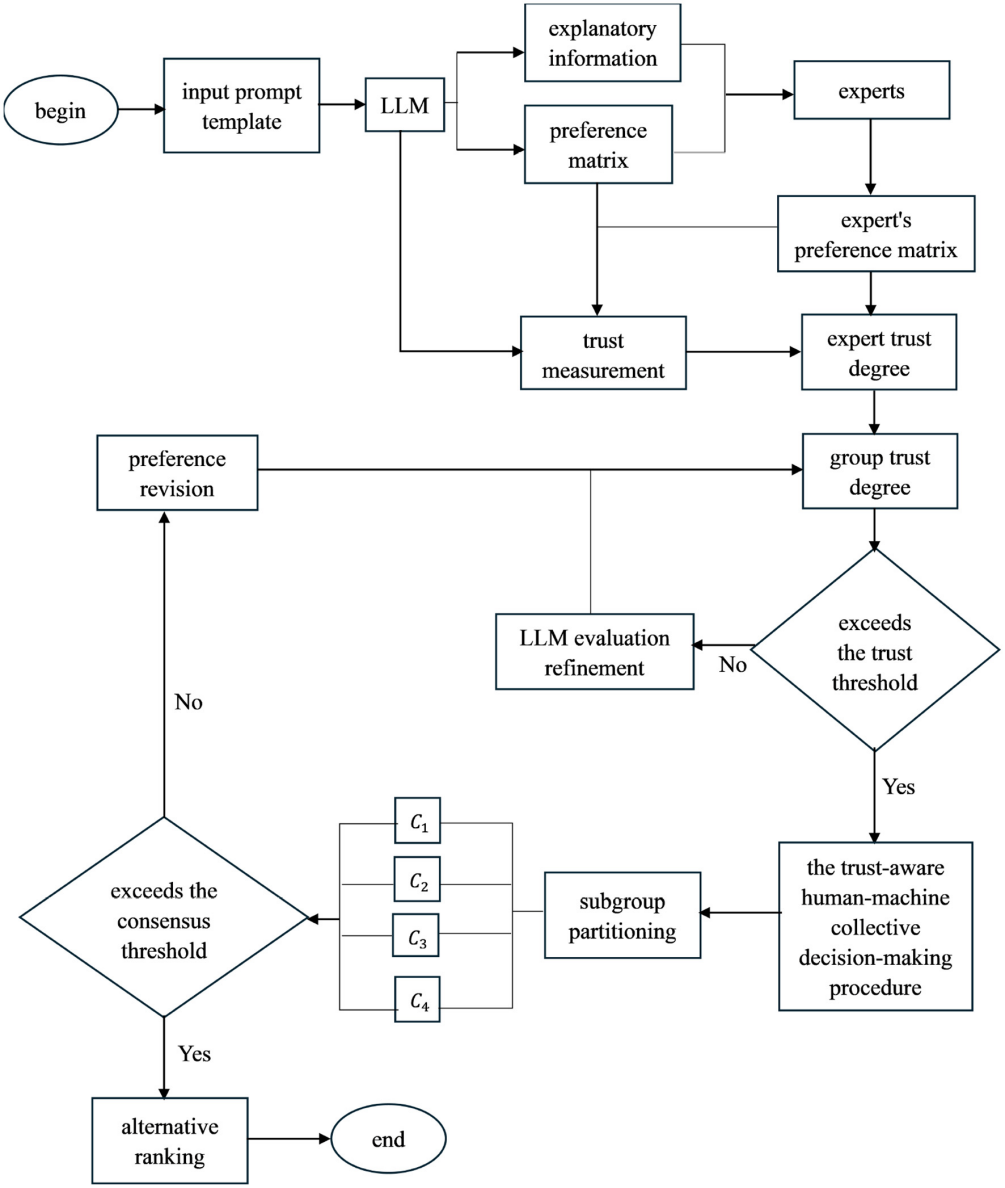
Human-AI group collaborative decision workflow.

The above process constitutes a human-AI collaboration decision-making closed-loop mechanism of “trust evaluation–opinion adjustment–consensus evolution,” and its overall workflow is shown in Figure 4.

## 5 Case analysis of human-AI collective collaborative decision-making

### 5.1 Case background

From July 17 to 21, 2021, continuous heavy rainfall occurred in Zhengzhou City and its surrounding areas in Henan Province. On July 20, public data from the Zhengzhou National Meteorological Station showed that the daily rainfall reached 624.1 mm, breaking the historical record for mainland China. During the period from 16:00 to 17:00, the hourly rainfall reached 201.9 mm, exceeding the urban drainage design standard by several times. The urban area was severely hit by flooding: major roads experienced extensive waterlogging, water depths in some residential communities and urban villages exceeded two meters, and residents' daily lives were severely affected. After the disaster, the Zhengzhou Municipal Flood Control and Drought Relief Headquarters activated a Level I emergency response on July 20 and organized experts from multiple departments, including emergency management, water affairs, transportation, public security, firefighting, housing and urban-rural development, and health, to form a joint emergency decision-making expert group. The group was tasked with collaboratively reviewing emergency response plans for urban waterlogging hazards and determining their priority sequence.

In this decision-making scenario, it is necessary to select the optimal combination strategy from multiple emergency response plans to minimize casualties and property losses. To simulate this human-AI collaborative decision-making process, this paper constructs a human-AI collaborative decision-making environment involving 20 experts and introduces a large language model (ChatGPT 4o) as a collaborative decision-making subject.

To ensure the validity of the experimental results, the following assumptions were made in advance:

- Expert behavior assumption: Each expert is rational and independent in the decision-making process. Their preference adjustments are based solely on their own judgment, LLM suggestions, and group feedback, without considering strategic gaming among experts.
- LLM output stability assumption: Under identical prompts and parameter settings, the evaluation information generated by the LLM is reproducible (achieved by fixing the temperature parameter and taking the mean of multiple samplings).
- Trust update assumption: Dynamic trust is determined solely by the normalized mean absolute deviation, ignoring the lag effects and nonlinear characteristics of trust.
- Subgroup division static assumption: In each iteration, subgroup division is based on the trust degree and consensus degree of the current round, assuming that experts do not change their basic behavioral types between rounds (e.g., from low trust to high trust).

Suppose there are 20 experts denoted as *E* = {*e*_1_, *e*_2_, …, *e*_20_}, who are required to make decisions on four alternative solutions based on specific circumstances. The alternatives are denoted as *X* = {*x*_1_, *x*_2_, *x*_3_, *x*_4_}, with the detailed content of each solution as follows:

*x*_1_ :Deploy the drainage system, strengthen water level monitoring, restrict passenger flow at subway stations, high-speed railway stations and other venues, evacuate people urgently, and implement urban traffic control.

*x*_2_ :Implement strict vehicle access control in key disaster-affected areas, strengthen material supply, and coordinate emergency and livelihood support.

*x*_3_ :Strengthen pipeline network dredging and dispatch of drainage pumping stations, open emergency shelters, and relocate residents affected in low-lying areas.

*x*_4_ :Prioritize ensuring the livelihoods of residents in severely affected areas, while organizing a team of drainage system technical personnel, building an optimized intelligent dispatch system to strengthen water level monitoring, and implementing refined drainage management in different regions.

Based on the characteristics of emergency management decision-making, the expert group determines four evaluation attributes and their weights: *c*_1_ : degree of waterlogging reduction (0.3), *c*_2_ : degree of remediation of residents' life and property losses (0.3), *c*_3_ : rapid recovery capacity of urban functions (0.2), *c*_4_ : human resource consumption (0.2).

Suppose there are 20 experts denoted as *E* = {*e*_1_, *e*_2_, …, *e*_20_}, who are required to make decisions on four alternative solutions based on specific circumstances. The alternatives are denoted as *X* = {*x*_1_, *x*_2_, *x*_3_, *x*_4_}, with the detailed content of each solution as follows.

### 5.2 Human-AI collaborative decision-making process

(1) Initial trust acquisition

**Figure 5:**
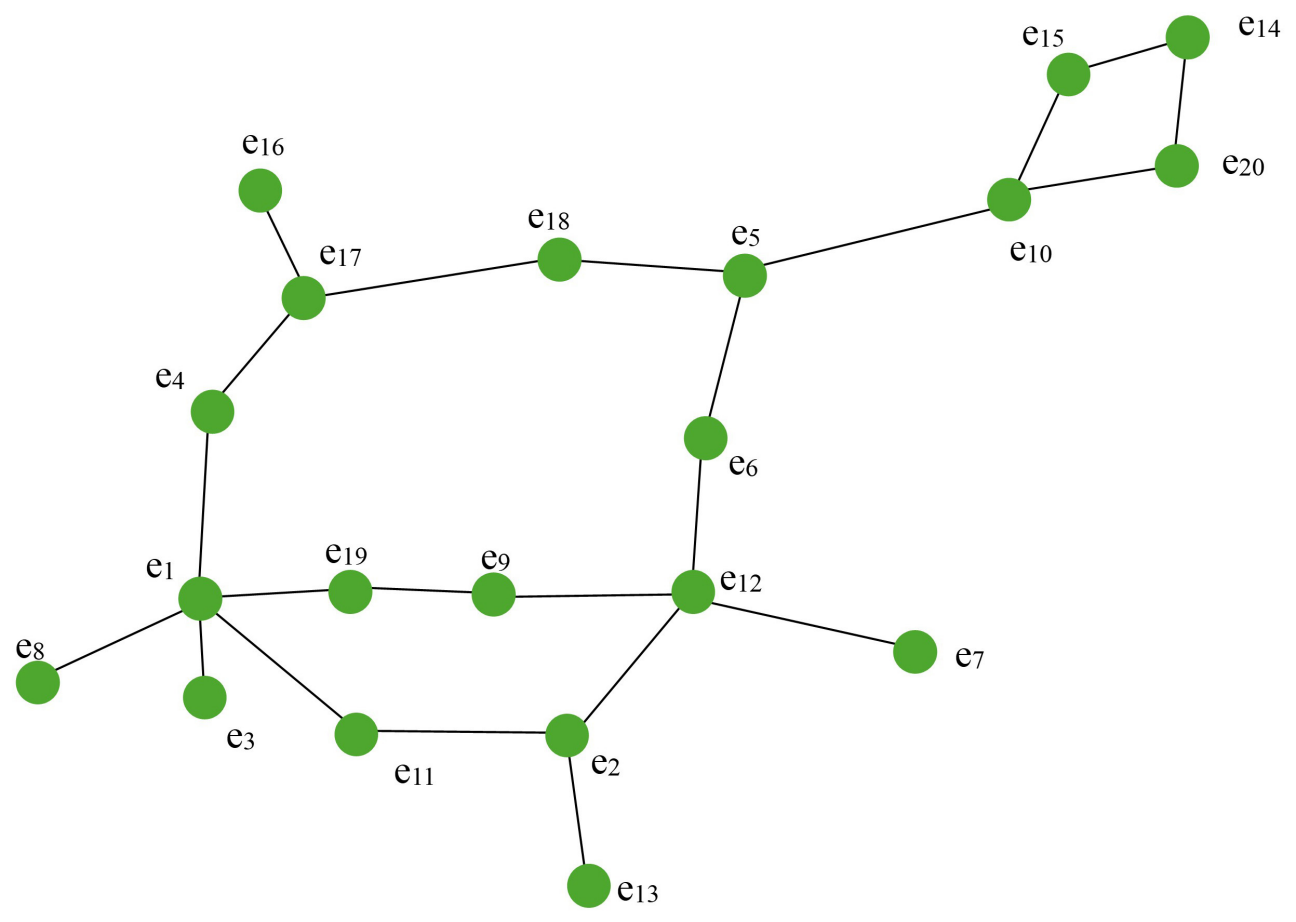
Expert-based social network analysis diagram.

First, the historical trust data of experts toward the large language model are obtained through the human-AI trust scale filled out by the experts. Combined with the social network relationships among experts, the trust transmission relationships between experts are calculated. Based on Equations 1–3, the initial trust level of each expert in the large language model is calculated. The social network structure of the experts is shown in Figure 5.

(2) Large language model generates evaluation information

According to the preset prompt template, the decision background information, alternative solutions, and evaluation attributes are input into the large language model, which then generates the evaluation values and explanatory information for each solution under different attributes. In this paper, ChatGPT is employed as the machine decision-making agent, and two main tasks are carried out. First, prompt engineering is used to guide ChatGPT in decision-making, which includes:

- Role setting: In the system prompt, the model is set as an emergency management domain expert.
- Domain knowledge injection: The background of the Zhengzhou “7.20” event, evaluation criteria, and rating rules are written into the prompt.
- Few-shot examples: Input-output examples of standardized scoring are provided to stabilize the output format and scoring standards.
- Parameter control: The temperature is set to 0 to ensure reproducibility, or multiple samplings are used to estimate variance.

Second, domain knowledge is injected based on RAG (Retrieval-Augmented Generation), which includes:

- Chunking and vectorizing the Zhengzhou rainstorm documents, emergency plans, and disaster reports, then storing them in a vector database (e.g., FAISS).
- During decision-making, relevant fragments are retrieved first and then spliced into the prompt before the model answers.

The evaluation results generated by ChatGPT are shown in Table 4, based on which the machine evaluation matrix is constructed, as shown in Table 5.

**Table 4:** LLM evaluation value and explanatory information.

Alternative	Attribute	Score	Explanation
*x*_1_	Degree of waterlogging reduction	0.7	By mobilizing drainage vehicles and pump stations for forced drainage, water accumulation on main roads can be quickly removed, restoring urban traffic capacity.
	Degree of remediation of residents' life and property losses	0.8	Emergency closure of affected subway stations and evacuation of passengers can effectively prevent secondary drowning incidents and ensure the safety of passengers' lives.
	Rapid recovery capacity of urban functions	0.7	The rapid activation of emergency shelters and the guarantee of supplies can maintain social stability and reduce casualties.
	Emergency resource and human resource consumption	0.6	Significant human resources need to be invested in dispatch management and facility repair.
*x*_2_	Degree of waterlogging reduction	0.6	Real-time water level monitoring and access control can effectively reduce casualties caused by vehicles mistakenly entering deep water areas.
	Degree of remediation of residents' life and property losses	0.7	Coordinating emergency and livelihood supplies can guarantee the basic living needs of residents in key disaster-affected areas.
	Rapid recovery capacity of urban functions	0.7	Strengthening material supply quickly stabilizes residents' lives, and enhanced control measures can prevent vehicles from entering dangerous areas.
	Emergency resource and human resource consumption	0.7	The consumption of coordinated supplies in the emergency rescue process is initially high but will stabilize later.
*x*_3_	Degree of waterlogging reduction	0.7	Strengthening pipeline network dredging and the dispatch of drainage pumping stations can systematically enhance the city's overall waterlogging drainage capacity.
	Degree of remediation of residents' life and property losses	0.7	Evacuation of residents and the opening of shelters can ensure the life safety of people in low-lying dangerous areas.
	Rapid recovery capacity of urban functions	0.6	The deployment of emergency supplies and livelihood support can reduce social panic and secondary risks caused by waterlogging.
	Emergency resource and human resource consumption	0.7	The dispatch of emergency teams and the guarantee of material supplies can maintain the basic living of relocated people and reduce the social impact of the disaster.
*x*_4_	Degree of waterlogging reduction	0.8	Intelligent dispatching of drainage resources can quickly lower the water level in the pipeline network and prevent waterlogging from further spreading to more areas.
	Degree of remediation of residents' life and property losses	0.8	Scientific dispatching of the drainage system can optimize the drainage sequence and maximize drainage efficiency under limited equipment conditions.
	Rapid recovery capacity of urban functions	0.7	Experience in refined dispatch of the drainage system can provide long-term data support for urban flood control planning.
	Emergency resource and human resource consumption	0.6	Drainage dispatching requires mobilizing a large number of mobile equipment and manual workers, resulting in significant resource consumption.

**Table 5:** Initial evaluation matrix for ChatGPT.

Alternative	*c*_1_	*c*_2_	*c*_3_	*c*_4_
*x*_1_	0.7	0.8	0.7	0.6
*x*_2_	0.6	0.7	0.7	0.7
*x*_3_	0.7	0.7	0.6	0.7
*x*_4_	0.8	0.8	0.7	0.6

(3) Experts generate preference matrix and trust update

After referring to the evaluation information and explanatory content generated by the large language model, the experts assess each alternative solution and form an expert preference matrix, as shown in Table 6 (see Appendix A for the complete table). Subsequently, Equation 4 is used to calculate the dynamic trust between the experts and the large language model, and the individual human-AI trust level is calculated by combining it with the initial trust. Considering that historical trust and social network trust are equally important in influencing initial trust, η is set to 0.5. In addition, since dynamic trust better reflects the real-time interaction state, the weight of initial trust is set to 0.3 in this paper. Finally, the individual trust levels of experts and the group human-AI trust level of 0.561 are obtained, as shown in Table 7 (see Appendix A for the complete table). Meanwhile, based on the expert preference matrix, the group preference matrix and the expert consensus levels are calculated, and the results are shown in Tables 8, 9, respectively.

**Table 6:** Expert initial preference matrix (selected rows extracted from source).

Expert	Alternative	*c*_1_	*c*_2_	*c*_3_	*c*_4_
*e*_1_	*x*_1_	0.8	0.7	0.6	0.9
	*x*_2_	0.9	0.9	0.7	0.8
	*x*_3_	0.7	0.8	0.7	0.8
	*x*_4_	0.6	0.4	0.8	0.8
…	…	…	…	…	…
*e*_3_	*x*_1_	0.9	0.5	0.7	0.8
	*x*_2_	0.8	0.7	0.5	0.6
	*x*_3_	0.9	0.8	0.4	0.6
	*x*_4_	0.5	0.6	0.6	0.8
…	…	…	…	…	…
*e*_19_	*x*_1_	0.4	0.9	0.7	0.5
	*x*_2_	0.6	0.9	0.8	0.8
	*x*_3_	0.5	0.9	0.3	0.7
	*x*_4_	0.6	0.8	0.6	0.8
*e*_20_	*x*_1_	0.9	0.5	0.9	0.7
	*x*_2_	0.4	0.8	0.6	0.9
	*x*_3_	0.7	0.8	0.8	0.8
	*x*_4_	0.9	0.9	0.5	0.9

**Table 7:** Initial human-AI trust level.

Expert	*HT*_*i*_	*ST*_*i*_	*INT*_*i*_	*DT*_*i*_	*T*_*i*_
*e*_1_	0.629	0.463	0.546	0.710	0.660
…	…	…	…	…	…
*e*_5_	0.557	0.559	0.558	0.531	0.539
*e*_6_	0.829	0.470	0.650	0.552	0.582
…	…	…	…	…	…
*e*_20_	0.871	0.469	0.670	0.644	0.652

**Table 8:** Initial group preference matrix.

Alternative	*c*_1_	*c*_2_	*c*_3_	*c*_4_
*x*_1_	0.7	0.7	0.7	0.6
*x*_2_	0.6	0.8	0.7	0.8
*x*_3_	0.7	0.8	0.5	0.6
*x*_4_	0.6	0.6	0.7	0.7

**Table 9:** Initial expert consensus level.

Expert	Consensus degree	Expert	Consensus degree
*e*_1_	0.737	*e*_2_	0.764
*e*_3_	0.745	*e*_4_	0.647
*e*_5_	0.635	*e*_6_	0.604
…	…	…	…
*e*_*n*_	0.681	*e*_20_	0.623

(4) Machine evaluation revision

To ensure the credibility of the human-AI collaboration decision-making results, the group trust threshold is set to γ = 0.6. As shown in Table 7, when the group human-AI trust level falls below 0.6, the large language model evaluation revision mechanism needs to be triggered. The large language model is guided through a prompt template to revise the original evaluation results. The revised machine evaluation matrix is shown in Table 10. After repeating the trust calculation process, the group trust level increases to 0.638, meeting the trust threshold requirement, thus entering the consensus reaching stage.

**Table 10:** Revised ChatGPT evaluation matrix.

Alternative	*c*_1_	*c*_2_	*c*_3_	*c*_4_
*x*_1_	0.75	0.75	0.7	0.75
*x*_2_	0.7	0.85	0.65	0.75
*x*_3_	0.7	0.8	0.6	0.6
*x*_4_	0.6	0.6	0.7	0.7

(5) Subgroup division

**Figure 6:**
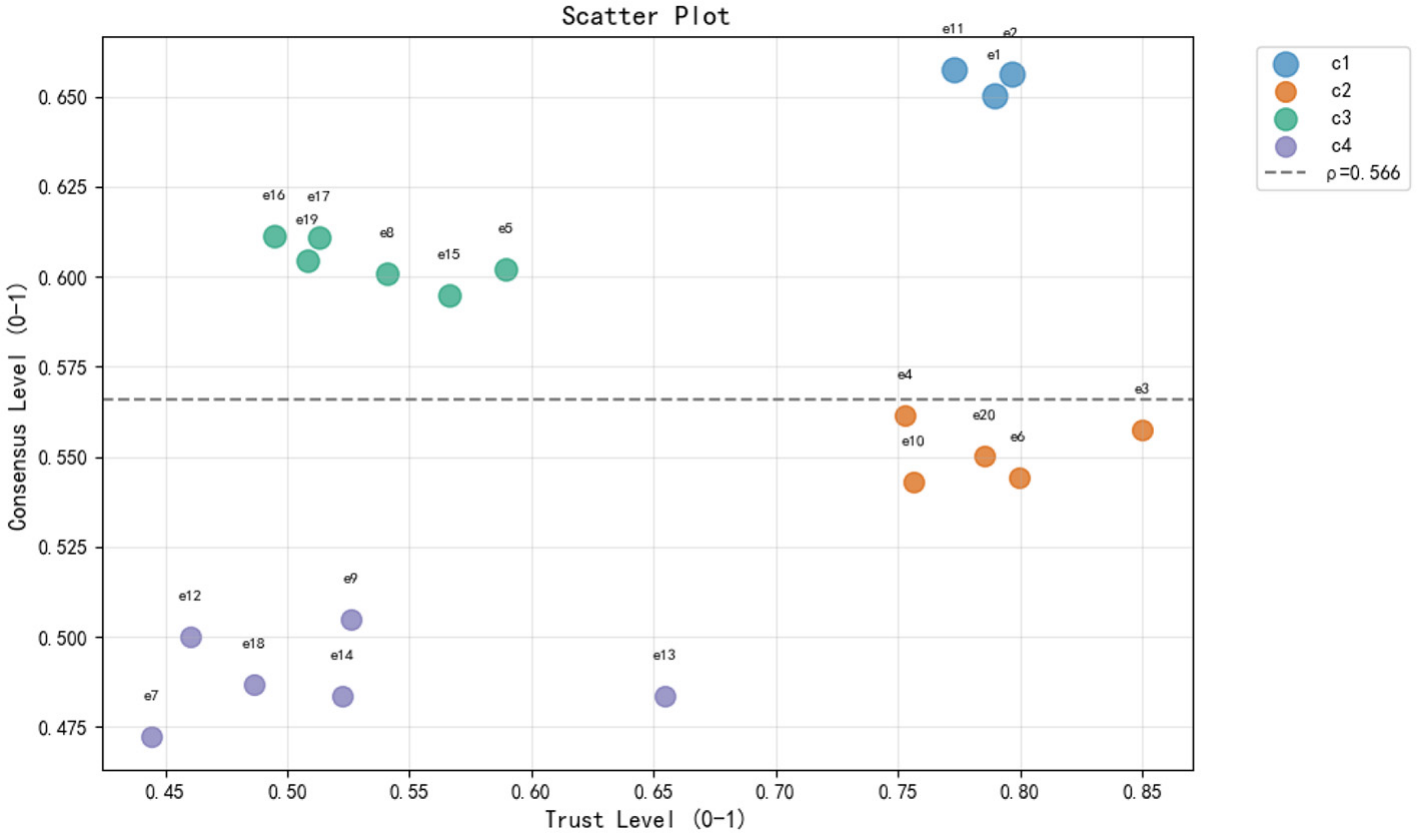
Subgroup division scatter plot.

Based on the two indicators of human-AI trust level and expert consensus level, the expert group is divided into subgroups by setting a trust threshold and a consensus threshold. The trust threshold is set to φ = 0.65, corresponding to a moderately high trust level, which helps identify experts who truly have high trust in the large language model and prevents excessive expansion of the high-trust group. The consensus threshold is set to ϕ = 0.7, representing a relatively high consistency level, which can effectively distinguish experts whose opinions are already close to the group opinion from those who need further coordination, avoiding imbalance in the division of high-consensus or low-consensus subgroups. Subgroup division is performed accordingly, and the subgroup consensus and group consensus are calculated using Equations 9, 10. The results are shown in Figure 6 (see Appendix A for the original consensus level data).

(6) Opinion adjustment and consensus evolution

To ensure the consistency and reliability of the decision-making results, the consensus threshold is set to σ = 0.8. In the first iteration, the group consensus level ρ does not reach the consensus threshold, triggering the opinion adjustment mechanism. For efficient subgroups and inefficient subgroups, their decision-making weights are adjusted through a weight reward and penalty mechanism. After expert discussion, the weight reward coefficient for the first round is determined as ζ^*t*^ = 0.2. According to Equations 11, 12, the weights of experts in efficient subgroups are increased by 0.01, while the weights of experts in inefficient subgroups are decreased by 0.005. For the trust subgroup, the direction of expert opinion adjustment is calculated using Equation 13, and the results are shown in Table 11.

**Table 11:** Trust subgroup expert opinion adjustment strategy.

Expert	*x*_1_	*x*_2_	*x*_3_	*x*_4_
*e*_1_	0.142	0.352	0.344	0.397
*e*_2_	0.273	0.410	0.199	0.489
*e*_3_	0.101	0.289	0.417	0.345
*e*_4_	0.241	0.302	0.516	0.207
*e*_5_	0.228	0.381	0.519	0.278

For the trust subgroup, in order to avoid conflicting opinions when experts modify their preferences autonomously, the directional deviation threshold is set to θ = 0.5. From Table 11, it can be seen that the adjustment directions of experts *e*_10_ and *e*_20_ for *x*_3_ exceed the threshold. Therefore, the opinion modifications of these two experts regarding *x*_3_ need to be adjusted, adopting the compromise principle mentioned earlier, where experts adjust their opinions along the median direction between their own opinions and the group preference direction. The management approach for preference modifications of experts within the proximate subgroup is as described in Section 3.5.

**Figure 7:**
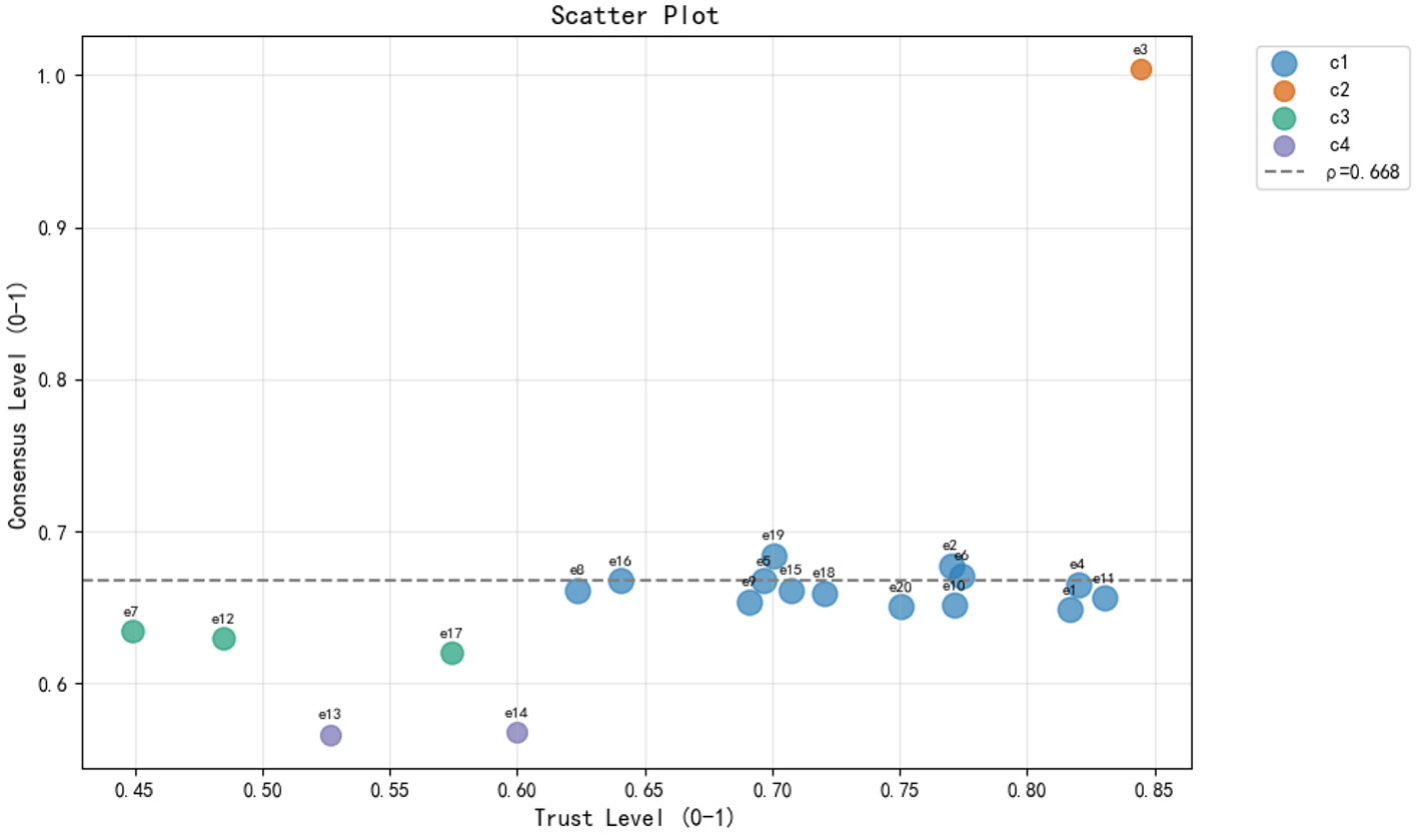
Scatter plot of the first-round update for subgroups.

After the first round of opinion management, the experts are re-divided into subgroups. The new subgroup division and group consensus level are shown in Figure 7. It can be seen that after the first round of adjustment, the group consensus still has not reached the threshold, so step five is repeated.

(7) Consensus reaching

**Figure 8:**
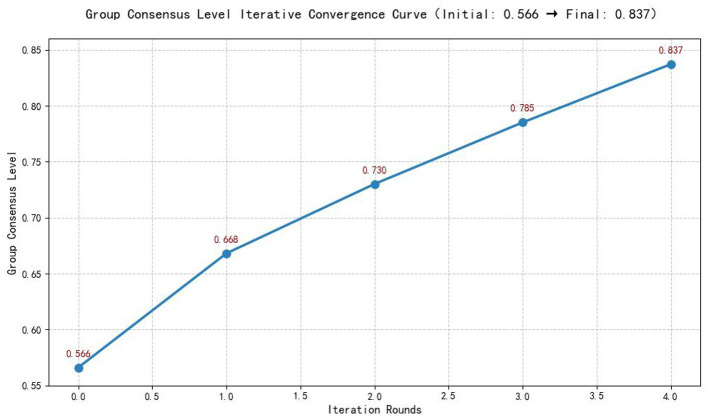
Convergence curve of the group consensus level.

After four rounds of opinion adjustment, the group consensus level reaches 0.837, exceeding the consensus threshold. The consensus iteration convergence curve is shown in Figure 8. Group consensus is achieved. The expert preference matrices are aggregated to obtain the final group preference matrix. Combined with the attribute weights *W* = {0.3, 0.3, 0.2, 0.2}, the comprehensive scores of each alternative are calculated as (0.700, 0.714, 0.692, 0.643). The alternative ranking is *x*_2_ > *x*_1_ > *x*_3_ > *x*_4_, and the optimal alternative is *x*_2_.

### 5.3 Comparative analysis

To verify the rationality and effectiveness of the proposed method, it is compared with two typical methods, including the traditional weight adjustment method and the reinforcement learning-based dynamic regulation method. The parameters such as expert weights and attribute weights in the traditional method (Xu et al., 2019, 2018) follow the settings in Section 5.1, and the comprehensive scores of alternatives obtained earlier are used as the comparison baseline. Meanwhile, the expert weights are adjusted through a unified rule to gradually improve the group consensus level.

In contrast, the reinforcement learning method models the group consensus reaching process as a Markov decision process and adopts a Deep Q-Network (DQN) for training. The process is detailed in Appendix B. In this method, the reinforcement learning agent selects weight adjustment strategies based on the current group consensus level and group trust state, and guides the improvement of the group consensus level through a reward function. The reward function of the reinforcement learning is designed as follows:


$$\begin{array}{l} r_{t}={\text{λ}}_{1}\left(GCL_{t}-GCL_{t-1}\right)+{\text{λ}}_{2}\left(GT_{t}-GT_{t-1}\right)-{\text{λ}}_{3}\left(Cost_{t}\right) \end{array}$$ (16)

Where *GCL*_*t*_ represents the group consensus level, *GTL*_*t*_ represents the group trust level, and *Cost*_*t*_ represents the expert opinion adjustment cost. To comprehensively evaluate the performance of different methods, this paper selects the following indicators for comparison:

(1) Number of consensus-reaching rounds, used to measure the efficiency of consensus convergence;

(2) Final group consensus level, used to measure the consistency of group opinions;

(3) Expert opinion adjustment cost, used to measure the degree of intervention in the consensus-reaching process;

(4) Alternative ranking stability, used to evaluate the consistency of decision results obtained by different methods.

The consensus threshold is also set to 0.8, and Table 6 shows the initial preference matrix of the 20 experts. The comparison results are shown in Table 12. It can be seen that when the consensus threshold is set to 0.8, the proposed method requires only four iterations to exceed the threshold, while the literature method requires six iterations to raise the consensus level to 0.801, demonstrating significantly better convergence speed. In contrast, the reinforcement learning-based dynamic regulation method adaptively adjusts expert weights through an agent and requires an average of 5 iterations to reach the consensus threshold in the experiment. Although it outperforms the traditional weight adjustment method in convergence efficiency, it is still slightly slower than the proposed method.

**Table 12:** Comparative results of consensus-reaching methods.

Comparison metric	Proposed method	Reference (Xu et al., 2019, 2018)	RL method	Reference (Xu, 2016)
Number of iteration rounds	4	6	5	6
Final consensus level	0.837	0.801	0.815	0.817
Total magnitude of expert opinion adjustments (cost)	1.28	2.21	1.96	2.04
Alternative ranking stability	95.1%	82.7%	87.3%	86%

In terms of consensus quality, the final consensus level achieved by the proposed method exceeds the threshold by 0.037, while the literature method exceeds it by only 0.001. This indicates that the proposed method can achieve higher-quality group consensus while meeting the threshold requirement, benefiting from the precise guidance of the human-AI trust mechanism on the consensus direction, thereby avoiding the consensus quality bottleneck caused by relying solely on weight adjustment.

In terms of opinion adjustment cost, the total magnitude of expert opinion adjustment using the proposed method is 1.28, while it reaches 2.12, when relying solely on weight adjustment to drive consensus. Although, the reinforcement learning method can reduce some ineffective adjustments through dynamic strategies, its overall adjustment magnitude still reaches 1.96. This difference indicates that by introducing the human-AI trust mechanism and subgroup differentiated regulation strategies, the proposed method can effectively reduce the redundancy of expert opinion adjustments, achieving better consensus outcomes at a lower intervention cost, which aligns with the efficiency principle of minimal intervention and optimal consensus in group decision-making.

Importantly, the stability of alternative ranking achieved by the proposed method across multiple iterations is 95.1%, which is 12.4 percentage points higher than the 82.7% of the literature method, and also significantly higher than the 87.3% of the reinforcement learning method. This indicates that the alternative ranking of the proposed method is less affected by random fluctuations in the iteration process, and the decision results possess better robustness and reliability, providing a more stable basis for subsequent decision implementation. Moreover, compared with the reinforcement learning method, which requires an additional model training process, the proposed method maintains high consensus quality while offering better explainability and lower computational complexity, making it more suitable for practical human-AI collaboration group decision-making scenarios.

This paper also presents a comparative analysis between the proposed human-AI collaborative decision-making consensus reaching method and the LLM-only decision-making approach. The detailed process of the LLM decision-making is provided in Appendix C of the Supplementary material. As a baseline for comparison, this paper employs the large language model as the sole decision-making agent to directly evaluate the four emergency response alternatives. Under five criteria-life safety protection, response timeliness, coverage breadth, resource feasibility, and systemic sustainability—the model generates an evaluation matrix in a single pass and produces a weighted ranking. The resulting preference order is *A* > *C* > *D* > *B*, with Scheme A (evacuation and traffic control) being recommended, as it achieves the highest scores in life safety and response timeliness. Although Scheme D exhibits the strongest systemic sustainability, it is ranked lower due to its reliance on medium-to-long-term system development and insufficient timeliness. Further weight sensitivity analysis shows that when the evaluation focus shifts from “immediate safety” to “long-term governance,” the preferred solution flips between A and C. This indicates that LLM-only decision-making is a one-time, single-point output, with the number of iteration rounds fixed at 1 and no group consensus reaching process (the consensus level metric becomes invalid and the opinion adjustment magnitude is zero).

Moreover, the ranking result is highly sensitive to the criterion weight settings, making stability difficult to guarantee. More importantly, the LLM-only decision ultimately fails to select the optimal solution, namely Scheme B (corresponding to Alternative 2), which is inconsistent with the actual situation. In contrast, the proposed method, through trust measurement and subgroup collaborative guidance, converges to high-quality consensus within a limited number of rounds even under conditions of disagreement among multiple agents, and significantly outperforms the LLM-only baseline in terms of ranking stability, opinion adjustment cost, and decision interpretability. While AI excels at rapid computation and ranking under predefined criteria, its understanding is often limited when it comes to the real-world constraints, implicit risks, policy contexts, interest balancing, and long-term governance needs involved in emergency decision-making. Human involvement can, to a certain extent, overcome such limitations by incorporating experiential knowledge and contextual awareness into the decision process and exercising quality control at critical junctures. Therefore, the human-AI collaboration proposed in this paper is better equipped to handle complex, dynamic, and ill-structured problems.

Meanwhile, as shown in Table 12, the human-expert-only decision-making methods, that is, those in references (Xu et al., 2019, 2018) and [Xu, 2016], perform worse than the proposed human-AI collaboration in terms of the number of iteration rounds and ranking stability. This is because natural disaster decision-making often involves multiple attributes and is accompanied by massive multi-source heterogeneous information (e.g., meteorological, hydrological, public opinion, and traffic data). Human experts have limited cognitive capacity and find it difficult to complete comprehensive information extraction and integration within a short time. Processes such as organization and coordination, information synchronization, and opinion aggregation consume considerable time and human resources. Therefore, integrating AI into traditional decision-making methods can improve efficiency and robustness to a certain extent, and better meet the requirements of natural disaster decision-making.

### 5.4 Sensitivity analysis

(1) Group trust threshold γ = 0.6

The group trust level represents the average trust of experts in the LLM's evaluation information. If it falls below 0.6, it indicates that the majority of experts are skeptical about the LLM's output. In such a case, directly using the LLM's evaluation would reduce the credibility of the decision, and a correction mechanism needs to be triggered. The value 0.6 is slightly above the random level of 0.5, representing a moderately high level of trust, which avoids being either too sensitive (frequent corrections) or too sluggish (allowing a low-trust state to persist).

**Figure 9:**
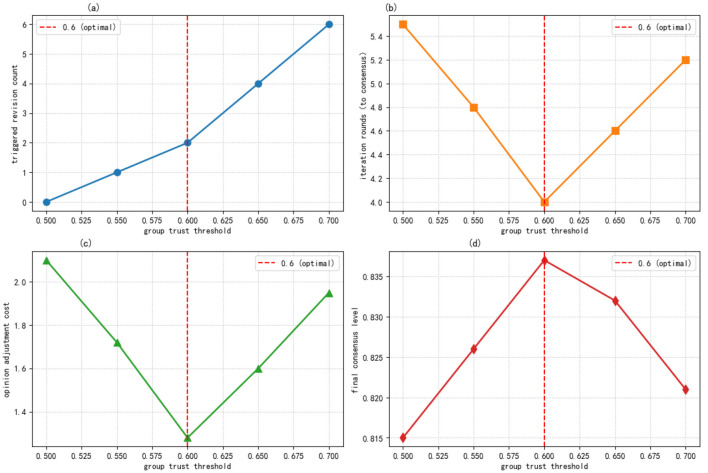
Convergence curve of the group consensus level. **(a)** LLM evalution revision trigger count, **(b)** number of iteration round required for consensus convergence, **(c)** total cost of expert opinion adjustments, and **(d)** final group consensus level.

Sensitivity experiment design: Let γ take values in [0.50, 0.55, 0.60, 0.65, 0.70], with other parameters fixed. The evaluation is conducted using four indicators: number of correction triggers, number of iterations, adjustment cost, and final consensus level. The results show that when γ = 0.6, the number of corrections is moderate (two times), the number of iterations is the smallest, the cost is the lowest, and the consensus level is the highest. The results are shown in Figure 9.

(2) Trust division threshold φ = 0.65

The trust threshold is used to classify experts into high trust and low trust in the LLM, and this value should be higher than the group trust threshold to effectively distinguish the core group that truly relies on the LLM. In group decision-making, this threshold should be greater than the random trust level (0.5), but should not be set too high, as that would result in an excessively small proportion of high-trust experts. Meanwhile, in social network trust propagation, the threshold of 0.5 serves as the boundary between positive and negative propagation. Taking 0.65 as a moderately high level can both identify experts who genuinely trust the LLM and avoid over-screening.

**Figure 10:**
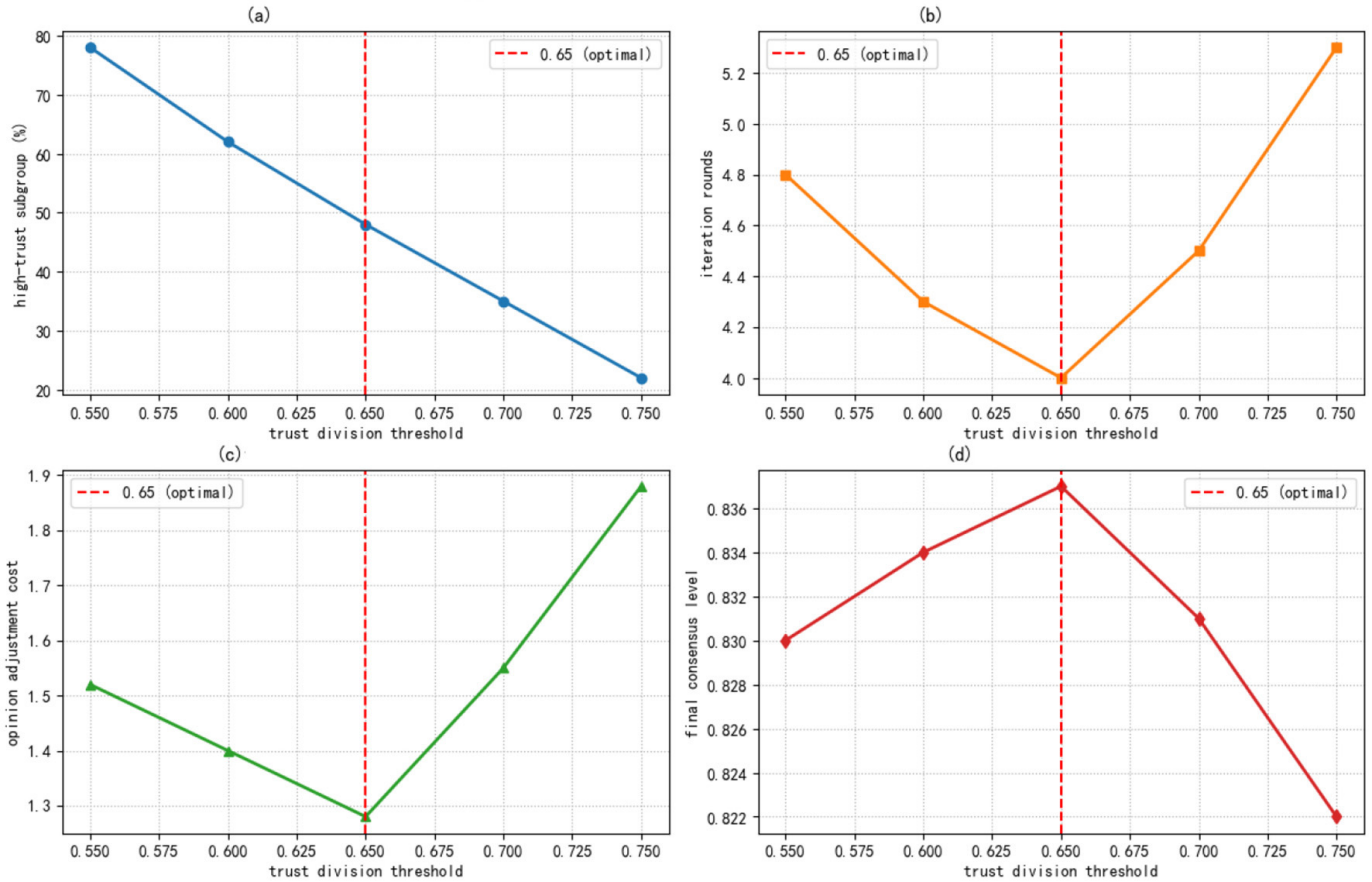
Convergence curve of the group consensus level. **(a)** High-trust expert (%), **(b)** rounds needed for consensus, **(c)** total expert adjustment cost, and **(d)** group consensus level.

Sensitivity experiment design: Let φ take values in [0.55, 0.60, 0.65, 0.7, 0.75], with other parameters fixed. The evaluation is conducted using four indicators: the proportion of high-trust experts, the number of iterations, the opinion adjustment cost, and the final consensus level. The results show that when φ = 0.65, the proportion of high-trust experts is close to 50%, the subgroup structure is balanced, and consensus convergence is optimal. The results are shown in Figure 10.

(3) Consensus division threshold ϕ = 0.7

The consensus degree refers to the similarity between an expert's preference and the group average preference. A value of 0.7 represents a relatively high level of consistency, indicating that the expert's opinion is within an acceptable distance from the group center. This value should be lower than the final consensus threshold, ϕ = 0.7 serving as an intermediate transition standard. If this value is too high (e.g., 0.75), only a few experts would be classified as high-consensus, resulting in an overly large low-consensus group and concentrated guidance pressure. If it is too low (e.g., 0.65), the majority of experts would be regarded as high-consensus, leading to insufficient identification of those who actually deviate from the group opinion.

**Figure 11:**
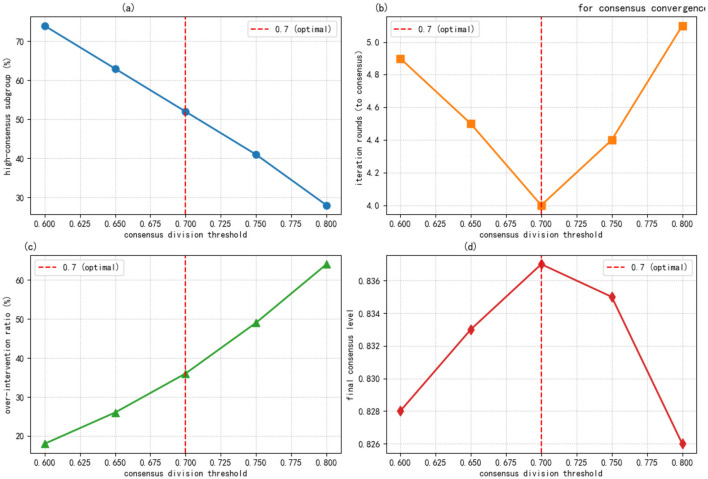
Convergence curve of the group consensus level. **(a)** High-consensus experts, **(b)** number of iteration round required for consensus convergence, **(c)** proportion of experts whose opinions are focibly modified, and **(d)** group final consensus level.

Sensitivity experiment design: Let ϕ take values in [0.60, 0.65, 0.7, 0.75, 0.8], with other parameters fixed. The evaluation is conducted using four indicators: the proportion of high-consensus experts, the number of iterations, the over-intervention ratio, and the final consensus level. The results show that when ϕ = 0.7, the proportion of high-consensus experts is about 50%, the intervention ratio is moderate, and the convergence speed is the fastest. The results are shown in Figure 11.

(4) Consensus reaching threshold σ = 0.8

In group decision-making, a consensus level of 0.8 or above is generally required to consider opinions as highly consistent and to terminate the iteration. The value 0.8 is a widely accepted strong consensus standard (e.g., most LSGDM studies adopt 0.8 or 0.85). A value below 0.8 (e.g., 0.75) may lead to insufficient decision quality, while a value above 0.8 (e.g., 0.85) would unduly increase iteration costs.

**Figure 12:**
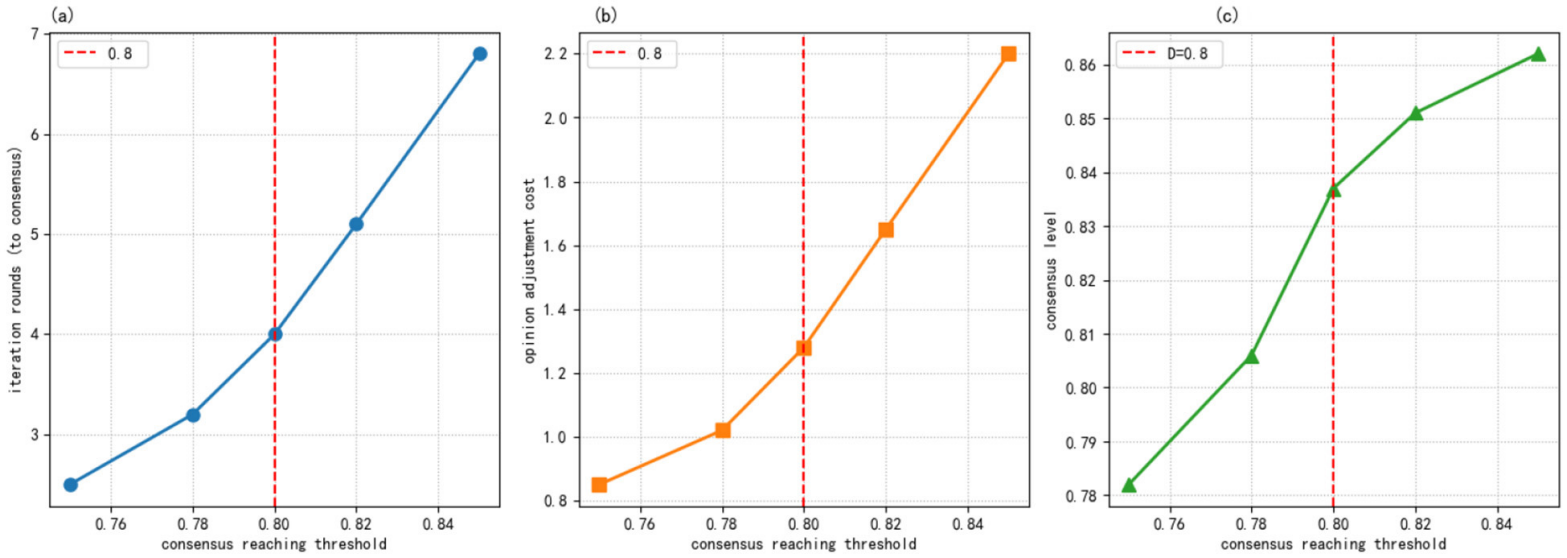
Convergence curve of the group consensus level. **(a)** iterations needed for consensus convergence, **(b)** total cost of expert opinion adjustments, and **(c)** final group consensus.

Sensitivity experiment design: Let σ take values in [0.75, 0.78, 0.80, 0.82, 0.85], with other parameters fixed. The evaluation is conducted using three indicators: number of iterations, adjustment cost, and final consensus level. The results show that when σ = 0.8, a balance between quality and efficiency is achieved, and the cost remains controllable, indicating that this setting is reasonable. The results are shown in Figure 12.

(5) Direction deviation threshold θ = 0.5

In the trust-based subgroup, if the angle between the expert's adjustment direction and the group consensus direction exceeds θ, a compromise adjustment is adopted; otherwise, the expert is allowed to adjust autonomously. The value 0.5 rad corresponds to approximately 28.6°, which falls within the moderate deviation range. A value that is too small (e.g., 0.3 rad) would force most experts to compromise, thereby weakening their autonomy; a value that is too large (e.g., 0.7 rad) would result in almost no compromise, thus losing the guidance effect. Based on the concept of acceptable deviation range in psychology, deviations within 30° are generally considered to be roughly aligned in direction and do not require mandatory intervention, whereas deviations exceeding 30° necessitate guidance. Therefore, 0.5 rad is a reasonable threshold.

**Figure 13:**
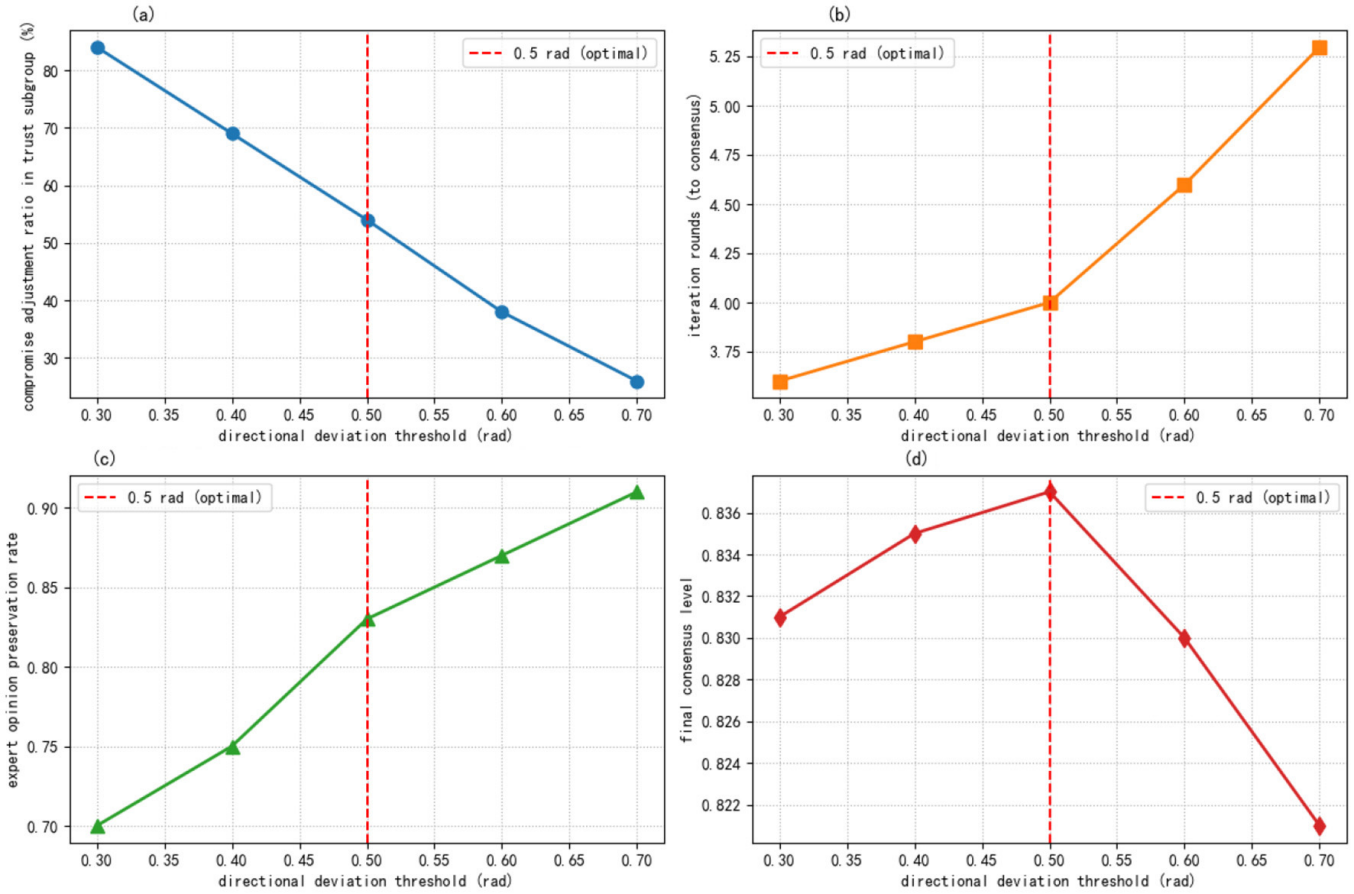
Convergence curve of the group consensus level. **(a)** Proportion of triggered compromise adjustments, **(b)** iterations needed for consensus convergence, **(c)** similarity of final preferences to initial preferences, and **(d)** group final consensus level.

Sensitivity experiment design: Let θ take values in [0.3, 0.4, 0.5, 0.6, 0.7], with other parameters fixed. The evaluation is conducted using the following indicators: angle of deviation, compromise adjustment ratio, number of iterations, and opinion preservation degree. The results show that when θ = 0.5 rad, the compromise ratio is approximately 54%, the number of iterations is the smallest, and the opinion preservation degree is moderate, representing the optimal balance point. The results are shown in Figure 13.

## 6 Conclusion

### 6.1 Research conclusion

This study constructs a dynamic trust-driven group consensus reaching framework for multi-source heterogeneous decision-making environments. In terms of trust measurement, it breaks through the limitations of traditional static trust assessment and proposes a two-dimensional human-AI trust model that integrates initial trust and dynamic trust, enabling dynamic characterization of the trust evolution process. Regarding the consensus reaching mechanism, expert groups are divided into subgroups based on the dual thresholds of “trust level and consensus level,” and differentiated opinion management strategies are designed for different subgroups: an autonomous negotiation model with minimal intervention is adopted for high-trust and high-consensus groups, while deep intervention of large language models is triggered for low-trust and low-consensus groups, achieving precise guidance by generating decision-making frameworks and conflict resolution plans. It demonstrates a closed-loop decision-making process from information generation, trust assessment, group division, opinion adjustment, to consensus convergence, and preliminarily explores the potential of large language models as collaborative decision-making subjects.

The empirical results from Zhengzhou, Henan Province, China, show that compared with traditional weight adjustment methods, the proposed method can significantly optimize the consensus reaching process. When the consensus threshold is set to 0.8, the average number of consensus-reaching iterations is reduced by 33.3%, the total magnitude of expert opinion adjustments is reduced by 69.5%, and the alternative ranking consistency is improved by 13.8%. These results demonstrate that the proposed method has significant advantages in improving consensus efficiency, reducing intervention costs, and enhancing decision stability.

### 6.2 Research limitations and future work

There are still many limitations in the paper that warrant discussion, as detailed below. Single case validation: this paper conducts experiments solely based on the Zhengzhou “7.20” rainstorm and waterlogging emergency decision-making scenario. Although this case is typical, a single scenario cannot demonstrate the generalizability of the method across more domains. Future work should validate the method in more types of decision problems (e.g., public health events, workplace safety accidents) and with different scales (number of experts, number of alternatives, and number of attributes).

Limited baseline comparisons: this paper only compares the proposed method with traditional weight adjustment methods and deep reinforcement learning methods. In fact, in recent years, various other baseline methods have emerged, such as blockchain-based consensus mechanisms and consensus models based on social network analysis. Due to space constraints, this paper does not provide a comprehensive comparison. Future research will expand the baseline set to conduct more thorough performance evaluations.

Risk of LLM's inherent biases: although, the LLM used in this study (ChatGPT) is a general-purpose model, its training data may contain regional, cultural, or value biases, which may indirectly affect decision outcomes through the generated evaluation information. This paper mitigates such biases to some extent through the expert feedback revision mechanism, but cannot completely eliminate them. It is recommended that, before actual deployment, multiple LLMs from different sources be used for cross-validation.

Threshold sensitivity: as mentioned above, the thresholds in this paper are set based on a specific case, and their optimality depends on the characteristics of the problem. Although, the sensitivity analysis shows that the method is robust around the threshold values, when the problem context changes substantially (e.g., when the trust distribution is extremely skewed), the thresholds may need to be recalibrated. Future research could explore adaptive threshold determination methods.

Lack of field testing: in this study, scholars in the field were selected as experiment participants, and all expert preference data were provided by these scholars rather than by experts from real emergency decision-making scenarios. Although, the data are based on a real case background and have been confirmed by domain experts, they still differ from actual human-AI interaction processes. Future research will recruit real experts to participate in human-AI collaboration experiments to test the effectiveness of the method in real-world environments.

Inaccuracy of trust measurement: this paper uses a seven-point Likert scale to measure historical trust. Although this scale has been validated in the literature, it is still subject to issues such as subjective bias and social desirability bias on the part of respondents. The calculation of social network trust relies on experts' self-reported network relationships, which may involve omissions or exaggerations.

At the same time, it should be noted that, in addition to the scale, the social network trust propagation model and the dynamic trust metrics, although based on mature methods in the existing literature, still lack independent empirical validation of their validity as indicators of actual human-AI trust behavior in the context of large language models. Readers should be aware of this limitation when interpreting the relevant results. Therefore, the human-AI trust measurement in this paper is only an approximation of real trust. In the future, behavioral experiments (e.g., trust games) or objective physiological indicators such as eye-tracking could be introduced to improve the accuracy of trust measurement.

It is also necessary to discuss the computational cost and overhead. Specifically the calculation of dynamic trust requires traversing *M*×*P* evaluation elements for each expert, with a complexity of (*N*·*M*·*P*); the subgroup division and the calculation of directional angles in trust-based subgroups also involve the same factor. When *N, M, P* all increase simultaneously, the time per iteration grows linearly. Although the number of LLM calls mainly depends on the number of iteration rounds and the frequency of trigger corrections, and has no direct relationship with the number of experts, the time for each generation of evaluation information increases with the length of the prompt (i.e., the number of alternatives and attributes). If the initial group trust level is low, multiple rounds of correction may be triggered, further increasing the cost of model calls. Meanwhile, the number of iteration rounds required to reach consensus increases with the initial degree of disagreement and the strictness of the consensus threshold, and the total computational overhead equals the per-round cost multiplied by the number of rounds, which may lead to significant resource consumption in cases with high thresholds or highly heterogeneous groups.

Future research can be further extended from the following aspects: (1) constructing a conflict management mechanism from the perspective of human-AI trust to address decision-making behavior conflicts that may arise during agent interaction; (2) further extending the existing consensus framework by introducing a dynamic LLM opinion feedback mechanism to strengthen its collaborative role in the group decision-making system, thereby promoting the application of human-AI collaboration decision-making mechanisms in complex situations; (3) in future work, behavioral experiments will be conducted to validate the predictive validity of the trust measures proposed in this paper, by recording objective behavioral indicators such as participants' actual adoption rate of LLM suggestions and their degree of reliance on the LLM. Meanwhile, multiple methods—including trust games and eye-tracking—will be introduced for cross-validation, to enhance the objectivity and reliability of trust measurement.

## Data Availability

The original contributions presented in the study are included in the article/Supplementary material, further inquiries can be directed to the corresponding author/s.
