# Supplementary material for: Trust-driven consensus reaching in human-AI hybrid large-scale group decision-making
Source: Front Artif Intell. 2026 Jul 29;9:1886098. doi: 10.3389/frai.2026.1886098 (PMC13461716; doi:10.3389/frai.2026.1886098)
Supplement: Supplementary file 1 [file Data_Sheet_1.pdf]

Supplementary Material

1 APPENDIX A

The complete data of the initial preference matrix of the 20 experts for the 4 alternatives are shown in Table S1 (corresponding to Table 6 in the main text). The complete data of initial human-AI trust are shown in Table S2 (corresponding to Table 7 in the main text). The expert survey data are shown in Table S3 of Appendix A (corresponding to the historical trust column in Table 7). The subgroup division data are shown in Table S4 of Appendix A (corresponding to Figure 6 in the main text).

Table S1: Expert initial preferences matrix (corresponding to Table 6 in the main text). Each expert provides preference values for 4 alternatives across 4 attributes c_1–c_4.

Expert	Alternative	c_1	c_2	c_3	c_4
e_1	x_1	0.8	0.7	0.6	0.9
	x_2	0.9	0.9	0.7	0.8
	x_3	0.7	0.8	0.7	0.8
	x_4	0.6	0.4	0.8	0.8
e_2	x_1	0.5	0.8	0.7	0.8
	x_2	0.8	0.7	0.9	0.6
	x_3	0.9	0.8	0.4	0.6
	x_4	0.5	0.6	0.6	0.8
e_3	x_1	0.9	0.5	0.7	0.8
	x_2	0.8	0.7	0.5	0.6
	x_3	0.9	0.8	0.4	0.6
	x_4	0.5	0.6	0.6	0.8
e_4	x_1	0.9	0.7	0.8	0.8
	x_2	0.7	0.8	0.6	0.9
	x_3	0.9	0.7	0.2	0.1
	x_4	0.7	0.7	0.9	0.6
e_5	x_1	0.8	0.8	0.8	0.5
	x_2	0.7	0.6	0.9	0.9
	x_3	0.7	0.9	0.5	0.3
	x_4	0.5	0.5	0.6	0.5
e_6	x_1	0.9	0.8	0.9	0.4
	x_2	0.5	0.7	0.5	0.9
	x_3	0.7	0.7	0.8	0.2
	x_4	0.8	0.6	0.7	0.6
e_7	x_1	0.3	0.5	0.6	0.3
	x_2	0.6	0.9	0.6	0.8
	x_3	0.5	0.9	0.2	0.3

Expert	Alternative	c_1	c_2	c_3	c_4
	x_4	0.5	0.6	0.5	0.8
e_8	x_1	0.5	0.5	0.9	0.7
	x_2	0.7	0.9	0.8	0.8
	x_3	0.5	0.9	0.6	0.2
	x_4	0.4	0.4	0.7	0.6
e_9	x_1	0.9	0.9	0.9	0.9
	x_2	0.7	0.7	0.8	0.8
	x_3	0.6	0.8	0.7	0.1
	x_4	0.5	0.6	0.8	0.9
e_{10}	x_1	0.9	0.8	0.6	0.8
	x_2	0.8	0.9	0.5	0.7
	x_3	0.7	0.8	0.8	0.9
	x_4	0.4	0.4	0.8	0.6
e_{11}	x_1	0.8	0.8	0.8	0.5
	x_2	0.6	0.8	0.8	0.8
	x_3	0.9	0.8	0.4	0.7
	x_4	0.6	0.5	0.9	0.6
e_{12}	x_1	0.4	0.7	0.9	0.9
	x_2	0.3	0.7	0.5	0.9
	x_3	0.9	0.8	0.2	0.7
	x_4	0.5	0.5	0.7	0.6
e_{13}	x_1	0.3	0.5	0.5	0.8
	x_2	0.8	0.9	0.6	0.6
	x_3	0.6	0.8	0.9	0.8
	x_4	0.6	0.9	0.8	0.9
e_{14}	x_1	0.4	0.7	0.7	0.3
	x_2	0.4	0.9	0.5	0.8
	x_3	0.9	0.9	0.3	0.9
	x_4	0.3	0.7	0.9	0.9
e_{15}	x_1	0.7	0.7	0.5	0.4
	x_2	0.5	0.9	0.8	0.7
	x_3	0.6	0.9	0.6	0.9
	x_4	0.6	0.5	0.5	0.9
e_{16}	x_1	0.6	0.6	0.8	0.8
	x_2	0.5	0.8	0.6	0.8
	x_3	0.4	0.9	0.7	0.8
	x_4	0.5	0.2	0.7	0.5
e_{17}	x_1	0.8	0.7	0.7	0.6
	x_2	0.6	0.9	0.7	0.8
	x_3	0.6	0.7	0.3	0.9
	x_4	0.4	0.6	0.5	0.9
e_{18}	x_1	0.6	0.9	0.8	0.8
	x_2	0.9	0.9	0.7	0.7
	x_3	0.4	0.9	0.8	0.4

Expert	Alternative	c_1	c_2	c_3	c_4
	x_4	0.4	0.3	0.7	0.9
e_{19}	x_1	0.4	0.9	0.7	0.5
	x_2	0.6	0.9	0.8	0.8
	x_3	0.5	0.9	0.3	0.7
	x_4	0.6	0.8	0.6	0.8
e_{20}	x_1	0.9	0.5	0.9	0.7
	x_2	0.4	0.8	0.6	0.9
	x_3	0.7	0.8	0.8	0.8
	x_4	0.9	0.9	0.5	0.9

Table S2. Initial human-AI trust level of 20 experts (corresponding to Table 7 in the main text).

Experts	HT_i	ST_i	INT_i	DT_i	T_i	GT
e_1	0.629	0.463	0.546	0.710	0.660	0.561
e_2	0.614	0.451	0.533	0.640	0.608	–
e_3	0.786	0.484	0.635	0.606	0.615	–
e_4	0.814	0.296	0.555	0.601	0.587	–
e_5	0.557	0.559	0.558	0.531	0.539	–
e_6	0.829	0.470	0.650	0.552	0.582	–
e_7	0.614	0.270	0.442	0.433	0.435	–
e_8	0.500	0.597	0.549	0.523	0.531	–
e_9	0.500	0.353	0.427	0.565	0.524	–
e_{10}	0.557	0.492	0.525	0.629	0.598	–
e_{11}	0.743	0.367	0.555	0.619	0.600	–
e_{12}	0.529	0.507	0.518	0.471	0.485	–
e_{13}	0.729	0.442	0.585	0.665	0.641	–
e_{14}	0.671	0.644	0.658	0.487	0.538	–
e_{15}	0.614	0.458	0.536	0.554	0.549	–
e_{16}	0.457	0.174	0.316	0.556	0.484	–
e_{17}	0.300	0.375	0.338	0.594	0.517	–
e_{18}	0.647	0.297	0.477	0.568	0.541	–
e_{19}	0.371	0.400	0.386	0.585	0.525	–
e_{20}	0.871	0.469	0.670	0.644	0.652	–

Table S3. Expert survey data used to compute historical trust T_{hist} (corresponding to the historical trust column in Table 7 of the main text). Scores on items Q1–Q10 use a 7-point Likert scale.

Expert	Q1	Q2	Q3	Q4	Q5	Q6	Q7	Q8	Q9	Q10	score	HT_i
e_1	4	5	4	4	5	4	5	4	4	5	44	0.629
e_2	4	4	4	4	5	4	5	4	4	5	43	0.614
e_3	5	6	5	6	5	6	6	5	5	6	55	0.786
e_4	6	6	5	6	6	6	6	5	5	6	57	0.814
e_5	4	4	4	4	4	4	4	4	3	4	39	0.557
e_6	6	6	6	6	6	6	6	5	5	6	58	0.829
e_7	4	4	5	4	4	4	5	4	4	5	43	0.614
e_8	3	4	3	4	3	4	4	3	3	4	35	0.500
e_9	4	3	4	3	4	3	4	3	4	3	35	0.500
e_{10}	4	4	4	3	4	4	4	4	4	4	39	0.557
e_{11}	5	5	5	5	5	5	6	5	5	6	52	0.743
e_{12}	4	4	4	4	4	3	4	3	3	4	37	0.529
e_{13}	5	5	5	5	5	5	5	5	5	6	51	0.729
e_{14}	5	5	5	4	5	4	5	4	5	5	47	0.671
e_{15}	4	4	4	4	4	5	4	5	4	5	43	0.614
e_{16}	3	3	3	3	3	4	3	4	3	3	32	0.457
e_{17}	2	2	2	2	2	2	2	3	2	2	21	0.300
e_{18}	4	4	5	4	5	4	5	4	5	5	46	0.647
e_{19}	3	3	2	3	3	2	3	2	2	3	26	0.371
e_{20}	6	6	6	6	6	6	6	6	6	7	61	0.871

Table S4. Subgroup division and consensus level (corresponding to Figure 6 in the main text).

	c_1	c_2	c_3	c_4
E	e_1, e_2, e_{11}	$e_3, e_4, e_6, e_{10}, e_{20}$	$e_5, e_8, e_{15}, e_{16}, e_{17}, e_{19}$	$e_7, e_9, e_{12}, e_{13}, e_{14}, e_{18}$
Consensus level	0.661	0.549	0.609	0.489
Group consensus		0.566		

2 APPENDIX B

To construct a comparative experiment using reinforcement learning, the group consensus reaching process is modeled as a Markov decision process (MDP), and a Deep Q-Network (DQN) is employed for training. The reinforcement learning agent dynamically adjusts expert weights to facilitate group consensus reaching. The specific process and parameter settings are as follows:

(1) State space

In the t-th round of decision-making, the system state is defined as:

$$s_t = [GCL_t,\ GT_t, \overline{CL}_t, \overline{T}_t, \rho_1,\ \rho_2,\ \rho_3,\ \rho_4, t] \tag{S1}$$

where GCL_t is the current group consensus level, GT_t is the group human-AI trust level, $\overline{\mathrm{CL}}_t$ is the average expert consensus level, $\overline{T}_t$ is the average expert trust level, $\rho_1, \ldots, \rho_4$ are the proportions of the four subgroups, and t is the current iteration round. The state dimension is 9 in total, which is kept at a relatively low level to help improve the stability of the reinforcement learning training process.

(2) Action space

Considering that in the proposed method, the expert group has already been divided into subgroups based on "trust level–consensus level", to maintain consistency in the comparative experiment, the reinforcement learning method also takes subgroups as the basic regulation objects, influencing the group decision-making process by adjusting the expert weights of each subgroup. The reinforcement learning agent adjusts the weights of the four types of subgroups in each round as follows:

$$\alpha_t = [\Delta w_{HE}, \Delta w_{HT}, \Delta w_{LC}, \Delta w_{LE}], \tag{S2}$$

Each adjustment takes discrete actions $\{-0.02,\ 0,\ +0.02\}$, where -0.02 means decreasing the subgroup weight, 0 means no change, and $+0.02$ means increasing the weight. Therefore, the size of the action space is $3^4 = 81$.

(3) Reward function

This paper takes the improvement of group consensus level as the main optimization objective, while also considering changes in group trust level and expert opinion adjustment costs. The reward function is set as:

$$r_t = \lambda_1 \cdot (\mathrm{GCL}_t - \mathrm{GCL}_{t-1}) + \lambda_2 \cdot (\mathrm{GT}_t - \mathrm{GT}_{t-1}) - \lambda_3\, \mathrm{Cost}_t, \tag{S3}$$

where GCL_t represents the group consensus level, GT_t represents the group trust level, and Cost_t represents the expert opinion adjustment cost. The weight for the consensus improvement term is set to $\lambda_1 = 0.6$; considering the auxiliary role of trust relationships in group decision-making, the weight for the trust improvement term is set to $\lambda_2 = 0.3$; a penalty term for adjustment cost is introduced to avoid excessive intervention, with its weight set to $\lambda_3 = 0.1$.

(4) Deep Q-Network

Regarding the selection of the reinforcement learning algorithm, this paper adopts the Deep Q-Network (DQN) as the comparative method. The main reasons for choosing DQN are as follows: the action space in this paper consists of discrete weight adjustment strategies, and DQN can effectively handle discrete action decision-making problems. Moreover, DQN has a relatively simple structure and stable training, making it suitable as a baseline method in comparative experiments. Furthermore, compared with more complex reinforcement learning algorithms such as PPO and DDPG, DQN has lower computational complexity, which helps highlight the efficiency advantage of the proposed method that does not require complex model training.

The network structure is as follows:

- Input layer: 9-dimensional state vector
- Hidden layer 1: 64 nodes (ReLU activation)
- Hidden layer 2: 64 nodes (ReLU activation)
- Output layer: 81-dimensional action values

The training parameters are shown in Table S5.

Table S5. DQN training parameters (Appendix B Tab. 1).

Parameter	Value
Learning rate	0.001
Discount factor	0.95
Buffer size	5000
Batch size	64
Target network update	every 200 steps

(5) Training settings

The training environment is consistent with the experiment described in Section 4.1 of this paper, i.e., the number of experts is set to 20, the number of alternatives is 4, the number of attributes is 4, the consensus threshold is 0.8, and the maximum number of decision-making rounds is 20. In terms of training parameters, the number of training episodes is set to 500, with a maximum of 20 decision-making rounds per episode. This setting is consistent with the maximum number of iterations for reaching consensus in the experiments of this paper, thereby ensuring consistency of the experimental environment. Meanwhile, preliminary experiments show that the reinforcement learning policy has largely converged after approximately 400 episodes; thus, setting the number of training episodes to 500 ensures model stability while avoiding overfitting. In addition, the initial exploration rate is set to 1, which decays by a factor of 0.995 gradually to 0.05, ensuring sufficient exploration capability in the early stage of training while gradually converging to a stable policy in the later stage.

(6) Experiment execution method

After training is completed, the learned policy is used to conduct 30 independent experiments, and the number of consensus-reaching rounds, final consensus level, expert opinion adjustment cost, and alternative ranking stability are recorded. The experimental results are averaged as the comparative results.
